# From Single-Core Nanoparticles in Ferrofluids to Multi-Core Magnetic Nanocomposites: Assembly Strategies, Structure, and Magnetic Behavior

**DOI:** 10.3390/nano10112178

**Published:** 2020-10-31

**Authors:** Theodora Krasia-Christoforou, Vlad Socoliuc, Kenneth D. Knudsen, Etelka Tombácz, Rodica Turcu, Ladislau Vékás

**Affiliations:** 1 Department of Mechanical and Manufacturing Engineering, University of Cyprus, 75 Kallipoleos Avenue, P.O. Box 20537, Nicosia 1678, Cyprus; krasia@ucy.ac.cy; 2 Laboratory of Magnetic Fluids, Center for Fundamental and Advanced Technical Research, Romanian Academy–Timisoara Branch, Mihai Viteazul Ave. 24, 300223 Timisoara, Romania; vsocoliuc@gmail.com; 3 Department for Neutron Materials Characterization, Institute for Energy Technology (IFE), 2027 Kjeller, Norway; Kenneth.knudsen@ife.no; 4 Soós Ernő Water Technology Research and Development Center, University of Pannonia, Zrínyi M. Str. 18., H-8800 Nagykanizsa, Hungary; tombacz@chem.u-szeged.hu; 5 Department of Physics of Nanostructured Systems, National Institute for Research and Development of Isotopic and Molecular Technologies, Donat Str. 67-103, 400293 Cluj-Napoca, Romania

**Keywords:** magnetic nanoparticle systems, ferrofluids, magnetic fluids, single core, multi-core, clusters, synthesis, functional coating, physical–chemical properties, structural characterization, magnetic characterization, small-angle scattering techniques, nanomedicine, biotechnology

## Abstract

Iron oxide nanoparticles are the basic components of the most promising magnetoresponsive nanoparticle systems for medical (diagnosis and therapy) and bio-related applications. Multi-core iron oxide nanoparticles with a high magnetic moment and well-defined size, shape, and functional coating are designed to fulfill the specific requirements of various biomedical applications, such as contrast agents, heating mediators, drug targeting, or magnetic bioseparation. This review article summarizes recent results in manufacturing multi-core magnetic nanoparticle (MNP) systems emphasizing the synthesis procedures, starting from ferrofluids (with single-core MNPs) as primary materials in various assembly methods to obtain multi-core magnetic particles. The synthesis and functionalization will be followed by the results of advanced physicochemical, structural, and magnetic characterization of multi-core particles, as well as single- and multi-core particle size distribution, morphology, internal structure, agglomerate formation processes, and constant and variable field magnetic properties. The review provides a comprehensive insight into the controlled synthesis and advanced structural and magnetic characterization of multi-core magnetic composites envisaged for nanomedicine and biotechnology.

## 1. Introduction 

Hybrid structures of colloidal nanoparticles designed for nanobiotechnology and nanomedicine [1,2,3,4,5,6,7,8], among them multifunctional magnetic nanoparticle–biomolecule–polymer hybrid systems with complex composition and topology [9,10,11,12], are receiving continuously increasing interest for medical diagnosis and treatment due to the newly acquired performances [13,14,15,16,17,18,19,20,21,22,23,24]. The required stability of superparamagnetic iron oxide nanoparticle (NP) systems in biological media target specific functionalities and selective drug delivery toward targeted locations [25,26,27,28,29] are ensured by molecular design of the dispersant/functional shell around the magnetic core [30,31,32]. Various interactions—van der Waals, electrostatic, molecular, entropic, hydrophobic, and magnetic—are contributing to the nanoscale self-assembly of nanoparticles [33,34,35,36,37,38,39,40,41,42,43] and provide colloidal nanoparticle clusters [44,45], in particular magnetoresponsive nanocomposite particles by merging magnetic and polymer materials with new collective properties, such as enhanced long-term stability and magnetic field-driven functionalities [43,46,47,48,49]. Among these, multi-core composites built up by magnetic nanoparticles embedded in non-magnetic matrices offer a composition, size, and structure dependent, sometimes nonlinear response to a constant or time-varying magnetic field [50,51,52,53,54,55,56]. In this way, a large variety of carefully engineered magnetoresponsive particles manufactured over time proved to be highly promising for nanomedicine, magnetic cell sorting, magnetic separations in biotechnology and environment purification, actuation, or catalysis [47,57,58,59,60,61,62,63,64,65,66,67,68,69,70,71,72,73,74].

It is essential to evidence that in contrast with agglomerates of single-core particles encountered in ferrofluids having weak colloidal stability, multi-core particles are the result of assembling a number of cores within a matrix. The number of cores in a multi-core particle is not changing with time, even in a magnetic field or intense shearing. The packing density, i.e., the distance between cores and also the size of the cores determines the intensity of magnetic interactions within the system and, finally, the cooperative magnetic behavior of multi-core particles [75,76,77]. Compared with single magnetic nanoparticles, the multi-core magnetic nanoparticles with a higher magnetic moment afford a considerable enhancement of the magnetic response [44,78,79], providing a significant driving force needed by most of the applications mentioned above. In order to assess the magnetic targeting/fixing applicability of magnetic particles, the magnetic moment of the particles is more relevant than mass magnetization [80,81].

In the above context, it is worthwhile mentioning that “bio-ferrofluids” with single and multi-core [82,83] or mostly multi-core magnetic particles (also commercial products) [84,85] extended significantly the category of “true” ferrofluids, containing practically only single-core magnetic nanoparticles dispersed in the carrier liquid. Among others, the addition of multi-core magnetic particles into a single-core ferrofluid reduces the long-term colloidal stability and changes completely the flow behavior in the magnetic field of the initially Newtonian ferrofluid [86]. To ensure kinetic stability and to avoid spontaneous aggregation in biorelevant media (for certain pH, salt, and protein concentration values) optimized and application determined surface coating (e.g., with polyelectrolytes) of iron oxide NPs is required for advanced bio-ferrofluid products manufactured for in vivo usage [87,88]. IONP’s surface chemistry and different coating/functionalization strategies to enhance the colloidal stability of bio-ferrofluids, involving natural polymers (dextran, chitosan, alginate), synthetic polymers (PEG, poly(ethylene glycol); PVP, poly(vinylpyrrolidone); PVA, poly(vinyl alcohol); PAA, poly(acrylic acid), etc.), dendrimers, dendrons, silanes, non-porous and porous silica, were thoroughly evaluated by Felder-Flesch and collaborators, including the relationship between coating and magnetic properties [89]. Colloidally stable ferrofluids with organic or aqueous carriers, in particular the single-core magnetic nanoparticles in their composition, proved to be a highly versatile and well-defined primary nanomaterial for manufacturing controlled magnetoresponsive superstructures of various morphologies [55,90,91,92,93,94]. In addition, nanostructures are assembled using the magnetostatic interaction between effectively diamagnetic and paramagnetic particles within a magnetized ferrofluid [35,95,96], the “magnetic holes” mechanism [97], allowing for a reversible assembling–disassembling process of practically non-magnetic particles [98] and also for the label-free manipulation and separation of cells and microorganisms using a ferromicrofluidic platform [99]. The magnetic hole mechanism was extended to the magnetic assembly of diamagnetic and magnetic particles immersed in a ferrofluid considered as a tunable magnetic continuum that controls the interactions between particles of different magnetization and sizes [100]. Specially designed composite spheres with embedded monodispersed micromagnets in a suspension—described as magnetic suspensions with shifted dipoles [101]—can self-organize in well-defined reconfigurable multi-core structures by simple magnetostatic interactions [102,103].

Among the remotely controlled endogenous (pH variation, enzymes etc.) or exogenous (e.g., light, temperature, electric field, magnetic field) stimuli-responsive nanoassemblies, designed to ensure dosage, spatial, and temporal controllability, the magnetic field driven bio-nanocomposites attracted tremendous scientific and technological interest [6,43,104]. In this context of magnetism-based nanomedicine and biotechnology, we will focus mainly on the *ferrofluid-based generation of multi-core magnetic nanocomposites*, which are motivated by the relevance and maturity of magnetic fluids technology in providing large quantities of high-performance ferrofluids for various biomedical and engineering applications [105,106,107,108,109,110,111]. In addition to numerous assembly procedures for different shapes of multi-core magnetic particles with an application-specific design of composition and functionalization, the paper presents the results of advanced characterization methods (transmission electron microscopy, TEM; scanning electron microscopy, SEM; high-resolution electron microscopy, HRTEM; dynamic light scattering, DLS) and zeta potential, X-ray, and neutron scattering techniques (small-angle x-ray scattering, SAXS; small-angle neutron scattering, SANS; polarization analyzed SANS, PASANS; very small-angle neutron scattering, VSANS; neutron reflectometry, NR; and magnetometry) and discusses magnetic properties in a constant and variable magnetic field, as well as particle structure (size, polydispersity, stabilizing shell thickness, composition of particle core and shell), magnetic structure (magnetic size and composition), particle interaction (interparticle potential, magnetic moment correlation, phase separation), cluster and supraparticle formation (developed aggregation and chain/bundle formation).

## 2. Multi-Core Superparamagnetic Nanospheres and Microspheres

### 2.1. Emulsion Procedures

Magnetic emulsions [112,113] composed of ferrofluid droplets dispersed in a non-miscible liquid can be successfully turned into superparamagnetic nanocomposite particles, usually of spherical shape. The controlled clusterization of magnetic nanoparticles using the miniemulsion technique [90,114,115,116], followed by encapsulation of the densely packed magnetic clusters in a polymer shell [62,117,118], is a successful joining of ferrofluid technology and emulsion procedures to provide highly magnetoresponsive multi-core particles [45,119,120,121,122,123,124,125]. This two-step colloidal assembly process has several important advantages [126]: (a) high-quality MNP building blocks can be produced by wet-chemical synthetic procedures of ferrofluids; (b) the MNPs are kinetically stable in the ferrofluid, and their clustering is initiated by some external trigger depending on the nature and surface chemistry of MNPs; and (c) size-controlled MNP clusters can be prepared if the MNP–MNP interaction potentials are engineered in an appropriate fashion. It is important to emphasize that MNP cluster formation is closely related to ferrofluid colloidal stability, i.e., the absence of aggregates in the ferrofluid used as primary nanomaterial is an essential feature [127].

**Figure 1:**
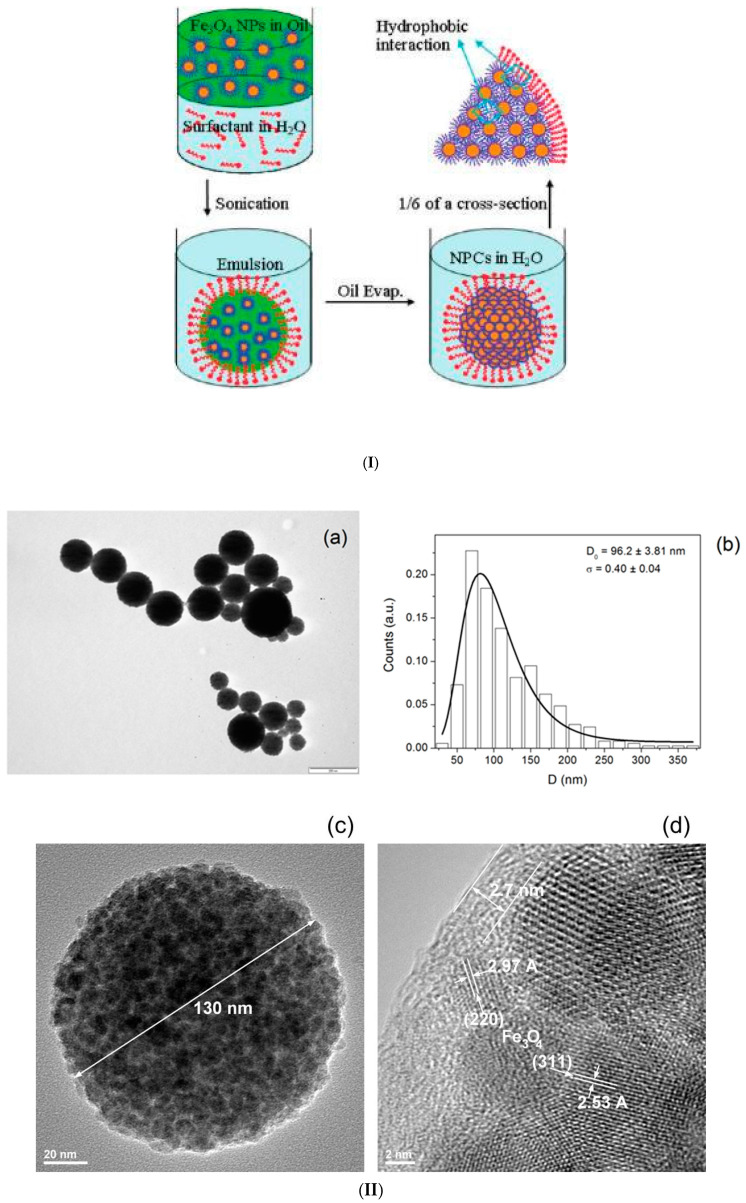

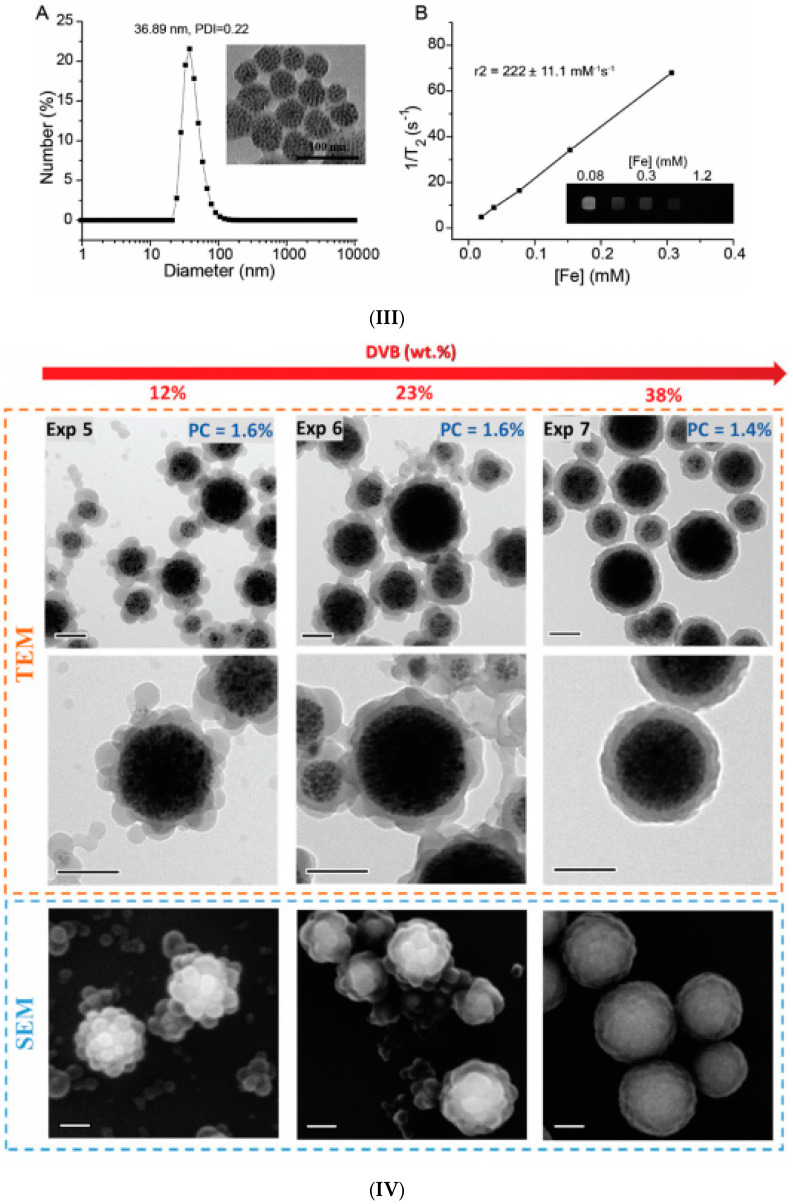

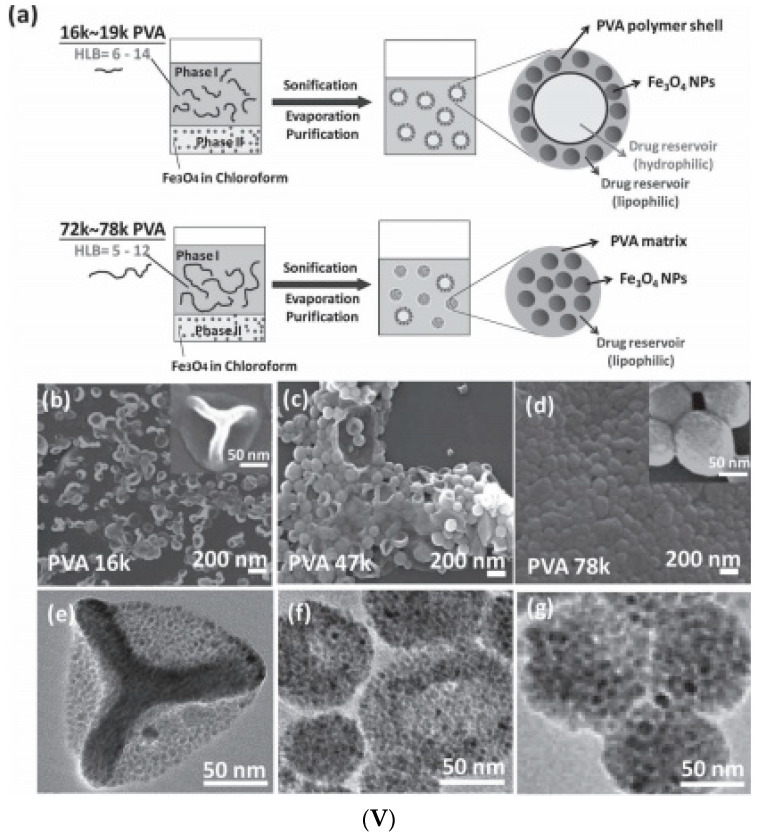
Magnetic nanoclusters prepared by emulsion procedures. (**I**) Preparation of clusters of magnetic nanoparticles: schematic of the oil-in-water miniemulsion procedure using hexane (or toluene)-based ferrofluid (Reprinted with permission from [122]. Copyright 2010 American Chemical Society); (**II**) Magnetic nanoparticle clusters stabilized with SDS: (**a**) TEM image (the scale bar is 200 nm); (**b**) the diameter distribution. (**c**) HRTEM image of magnetic clusters coated with polyacrylic acid; (**d**) HRTEM image of magnetic clusters coated with pNIPA (poly(N-isopropylacrylamide)–pAA (polyacrylic acid) (republished with permission of Royal Society of Chemistry, from [127]; permission conveyed through Copyright Clearance Center, Inc.); (**III**) (**A**) Dynamic light scattering and transmission electron microscopy image (insert) of PpIX-coated SPION nanoclusters; (**B**) Magnetic resonance (MR) relaxometry measurements of nanoclusters. An MR phantom image (inset) of nanoclusters at various concentrations in a microplate was also collected (republished with the permission of John Wiley and Sons, from [130]; permission conveyed through Copyright Clearance Center, Inc.). (**IV**) TEM and SEM micrographs of amphiphilic block copolymer (PDMAEMA) stabilized magnetic latex particles synthesized by a seeded semi-batch emulsion polymerization of styrene in the presence of increasing amounts of DVB: 12 wt %, 23 wt %, and 38 wt % (based on overall monomer mass), introduced either in the initial load or in both the initial load and the feed. Scale bar: 100 nm (republished with permission of Royal Society of Chemistry, from [131]. (**V**) Single emulsion particles (SEP) and double emulsion capsules (DEC). Primary material: chloroform-based Fe_3_O_4_ ferrofluid (**a**) Schematic of one-step emulsion synthesis incorporating iron oxide (IO) nanoparticles and single-component polymer poly(vinyl alcohol) (PVA). PVA with a MW of 16,000 and 19,000 g/mol gives double emulsion capsules, PVA with a MW of 72,000 and 78,000 g/mol gives single emulsion particles (SEP), PVA with a MW of 23,000 to 67,000 g/mol gives mixtures of both types. SEM and corresponding TEM images of dried DEC-IO and SEP-IO are shown in (**b**,**e**) for PVA-16k, (**c**,**f**) for PVA-47k, and (**d**,**g**) for PVA-78k. (Republished with permission of John Wiley and Sons, from [132]; permission conveyed through Copyright Clearance Center, Inc.).

The formation of ferrofluid droplets in emulsion followed by solvent evaporation to trigger nanoparticle clustering is a facile process to obtain closely packed and size-controlled clusters of magnetic NPs [128]. The basic procedure is represented schematically in Figure 1I [122]. To exemplify, a small volume of hexane-based ferrofluid (200 μL, concentration varies from 1 to 100 mg/mL) was added to 4 mL of 0.1 M CTAB (cetyl trimethylammonium bromide) aqueous solution. Then, all of the liquid was gently mixed by handshaking, followed by sonication for 2 min to form a stable micelle suspension. Afterwards, the mixture was heated in an 80 °C water bath and stirred at 500 rpm for 5 min so that the majority of the hexane was evaporated. Alternatively, hexane evaporation can be done by stirring under ambient conditions for several hours. Then, the solution was removed from the heat and stirred under a vacuum for 30 min to completely remove the hexane. By finishing solvent evaporation, the interparticle van der Waals attractions increase and the particles stick to one another, forming densely packed spherical nanoparticle clusters (NPC) [55,119,129].

Once the oil phase (hexane or toluene) is left to evaporate (water is added to keep the volume constant), the primary droplets transform into clusters, while the solvent swollen micelles form a mixture of empty micelles and free surface-active agent (CTAB). Throughout the ripening process, the primary droplets maintain a constant number of MNPs, since the nanoparticles do not have high enough water solubility to diffuse from the primary droplet [123]. The controlled clustering of magnetic nanoparticles from ferrofluids allows tailoring the size and magnetic moment of the particles. Using a toluene-based ferrofluid containing oleic acid-coated Fe_3_O_4_ nanoparticles, high magnetization magnetic clusters were prepared by the oil-in-water miniemulsion method [127,133]. The ferrofluid was added to the aqueous phase containing the surfactant (SDS or CTAB), and the mixture was ultrasonicated for 2 min to obtain small stable droplets of magnetic fluid in water. Then, the as-prepared miniemulsion was heated at 100° C to remove the toluene and then carefully washed several times with a methanol–water mixture, magnetically separated, and redispersed in water. In a second step, these magnetic clusters coated with the surfactants SDS or CTAB were encapsulated into polymers.

The TEM image in Figure 1IIa evidences the closed packed spherical clusters of approximately 100 nm mean size (Figure 1IIb) prepared from a toluene-based ferrofluid. The magnetization curves of magnetic clusters at room temperature show no measurable hysteresis or coercivity, which is consistent with the superparamagnetic behavior. The saturation magnetization of magnetic clusters has relatively high values: M_S_ = 63.9 A·m^2^/kg for magnetic clusters stabilized with SDS and M_S_ = 76.7 A·m^2^/kg for magnetic clusters stabilized with CTAB. Figure 1IIc,d provides the HRTEM images of closely packed magnetite NP clusters with pAAc and pNIPA–pAAc functional polymer coatings, respectively. Anion exchange and cation exchange magnetic microgels manufactured using the SDS and CTAB stabilized clusters provided remarkable performances in High Gradient Magnetic Separation (HGMS) processes [127,134]. Protoporphyrin IX (PpIX)-coated SPION nanoclusters were formed in a highly reproducible microemulsion procedure [130] by dissolving PpIX and small hydrophobic SPIONs (physical diameter = 7.3 ± 1.0 nm) in toluene, adding this mixture to water, followed by sonication. The PpIX-coated SPION nanoclusters dispersed in water have an average hydrodynamic diameter of ≈37 nm with a polydispersity index (PDI) of 0.22 (Figure 1IIIA), which is in good agreement with the sizes of tightly packed spherical cores of SPIONs evidenced by transmission electron microscopy (TEM). The superparamagnetic nanoclusters showed enhanced T2-contrast (i.e., hypointensity) compared to the control samples (Figure 1IIIB inset). The same emulsification/solvent evaporation technique was applied using a commercial ferrofluid (fatty acid-coated magnetic nanoparticle powder (Ferrotec Co.) dispersed in toluene) to obtain iron oxide NP clusters used as seeds in a semi-continuous procedure of surfactant-free emulsion polymerization of styrene (with or without DVB (divinylbenzene), cross-linking agent) [131]. The resulting poly(2-dimethylaminoethyl methacrylate)-b-polystyrene (PDMAEMA-b-PS) amphiphilic block copolymer encapsulated magnetite nanoclusters have a well-defined core shell structure for a higher amount of DVB, as evidenced in SEM and TEM micrographs (Figure 1IV). The preparation of core–shell type, double emulsion capsules (DEC) usually requires a two-step emulsifying process. According to [132] (Figure 1Va), these capsules are manufactured by a surfactant-free one-step emulsifying process, in which the oleic acid coated iron oxide nanoparticles of a chloroform-based ferrofluid were acting as DEC stabilizers, while poly(vinyl alcohol) (PVA) acted as both the shell constituent and the surfactant. SEM and corresponding TEM images of dried double emulsion and single emulsion iron oxide composite particles (DEC-IO and SEP-IO) are shown in Figure 1Vb–e.

In addition, in an emulsion procedure involving a second generation and clinically used photosensitizer (chlorin e6) and toluene-based ferrofluid with OA-coated Fe_3_O_4_ nanoparticles, nanoclusters with an average hydrodynamic diameter of 96.38 ± 4.6 nm were manufactured for dual mode imaging and photodynamic therapy [135]. A chloroform-based γ-Fe_2_O_3_/Fe_3_O_4_ ferrofluid was used as the polymer solvent/oil phase in the emulsion solvent evaporation process (ESE) for manufacturing SPION/polymer hybrid particles, starting from an oil-in-water emulsion procedure [136]. The initial core sizes in the ferrofluid ranged from 4 to 15 nm, whereas the assembling process using polystyrene dissolved also in chloroform resulted in flower-shaped surfactant-stabilized polystyrene beads with sizes between 50 and 250 nm [137]. The magnetic ordering of single-core NPs accomplished by their close assembly within the nanoflower multi-core domains resulted in a significant enhancement of the specific absorption rate (SAR), in comparison to single domain iron oxide nanoparticles subjected to the same alternating magnetic field conditions.

**Figure 2:**
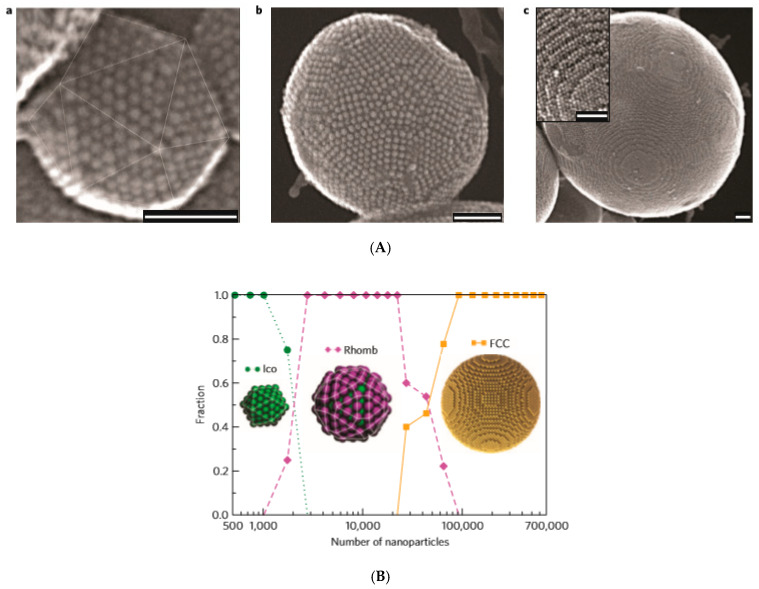
Entropy-driven supraparticle assembly. (**A**) Secondary electron scanning transmission electron microscopy (SE-STEM) images of typical supraparticles containing oleic acid coated cobalt–iron oxide nanoparticles initially dispersed in cyclohexane (core diameter 6 nm; hydrodynamic diameter 9 nm). (**a**) Supraparticle with a diameter of 105 nm with Mackay icosahedral symmetry, as indicated by the thin lines. (**b**) 216 nm supraparticle with anti-Mackay rhombicosidodecahedral structure. (**c**) 734 nm supraparticle consisting of a single face-centered cubic (FCC) crystal domain. Inset: a magnified view of the step edges of the FCC supraparticle. All scale bars are 50 nm. (**B**) Size dependence of the cluster structure–event-driven molecular dynamics (EDMD) numerical simulation. Structural transition from a Mackay icosahedron (Ico) to an anti-Mackay rhombicosidodecahedron (Rhomb) to a face-centered cubic (FCC) cluster, as observed for supraparticles consisting of nanoparticles. The fraction of structures, based on 121 supraparticles is plotted as a function of the number of nanoparticles per supraparticle. Fourteen icosahedra, 63 rhombicosidodecahedra, and 44 FCC clusters were observed. (Reprinted by permission from Copyright Clearance Center: Nature, Nature Materials, [138], Copyright 2015).

An oil-in-water miniemulsion procedure was applied to synthesize large magnetoresponsive supraparticles by entropy-driven clustering in a spherical confinement of oleic acid-coated cobalt–ferrite NPs of an apolar organic solvent (cyclohexane)-based ferrofluid [138], Figure 2A,B.

The cluster-size dependence in event-driven molecular dynamics (EDMD) simulations (Figure 2B) shows that the transitions from Mackay to an anti-Mackay to face-centered cubic (FCC) ordering approximately matched those shown in Figure 2A, which was observed experimentally. Entropy and spherical confinement proved to be sufficient for the formation of stable icosahedral MNP clusters without the contribution of interparticle attractive interactions. The same slow evaporation technique of emulsion droplets was applied to evidence that sharp cubic and rounded cubic NPs self-assemble also into spherical supraparticles [139].

In addition, large, micrometer-size superparamagnetic microparticles for affinity separation were synthesized in a four-step procedure using a hexane-based ferrofluid [120,140]: (1) creation of an oil-in-water emulsion in which OA-coated hydrophobic iron oxide nanoparticles of 5.3 nm mean physical size and a UV-activated initiator were distributed in hexane; (2) formation of uniform microparticles through emulsion homogenization and the evaporation of hexane; (3) functionalization of the microparticle with a PEG-functionalized surfactant and acrylic acid; and (4) polymerization of the microparticles.

The miniemulsion procedure proved to be useful to obtain magnetic nanocomposite particles with spatially separated functionalities [69,141] or to encapsulate magnetic nanoparticles together with fluorescent components [142,143].

**Figure 3:**
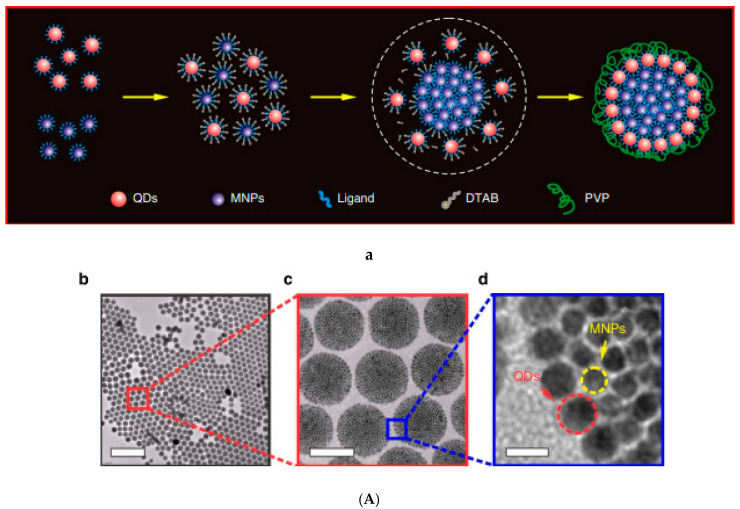

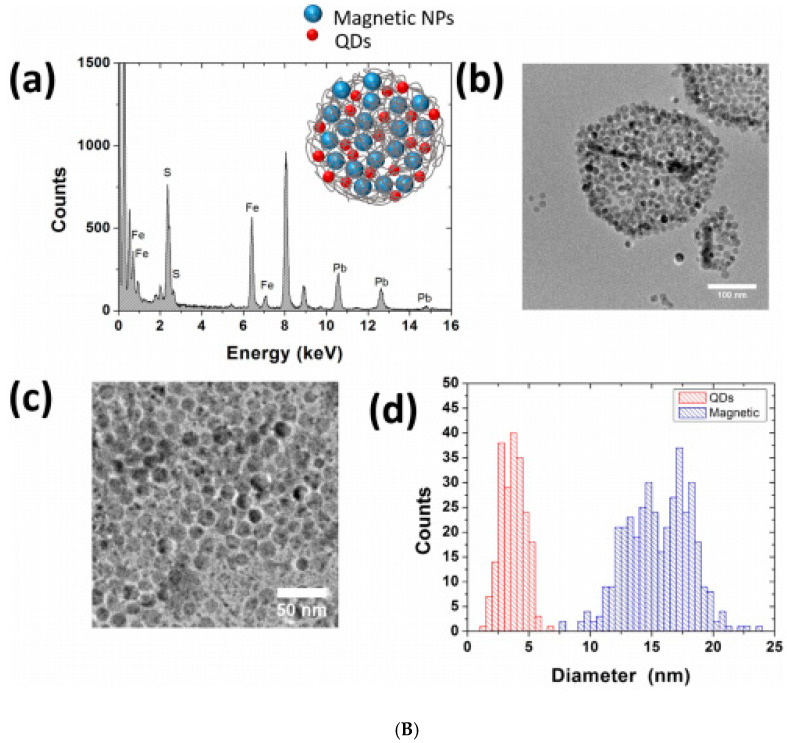
Co-assembling quantum dots and magnetic nanoparticles (NPs) of a ferrofluid in a magnetoresponsive core–shell nanostructure. (**A**) Synthesis and characterizations of core–shell-structured supraparticles (CS-SPs). (**a**) Schematic of the formation of the CS-SPs. A set of TEM images of CS-SPs at different magnifications. Scale bars, 500 nm, 100 nm, and 10 nm (**b**–**d**). Ferrofluid: magnetite NPs in chloroform carrier. Characteristic sizes: 9.0 nm for QDs (CdSe-CdS) and 5.9 nm for magnetite NPs (reprinted by permission from Copyright Clearance Center: Springer Nature, Nature Communications [144], Copyright 2014); (**B**) (**a**) Compositional analysis (EDX) of a PLGA nanostructure denoting the presence of elements of both magnetic IONPs and QDs (PbS). (inset) Schematic diagram of the magnetic NPs and QDs in the PLGA nanostructure. (**b**) TEM image of a typical PLGA nanostructure. (**c**) A detailed TEM image of a PLGA nanostructure revealing the presence of two types of NPs inside the structure. (**d**) Size distribution of both types of particles obtained from TEM images. The size distribution corresponds to the sizes of the magnetic NPs (15 nm) and QDs (4 nm). Ferrofluid: oleic acid-coated magnetite NPs in hexane carrier. (reprinted with permission from [145]. Copyright 2016 American Chemical Society).

An efficient procedure for assembling CdSe–CdS QDs with Fe_3_O_4_ MNPs into colloidal supraparticles (SPs) with a core–shell superstructure is given in [144]. In a typical synthesis process, 1 mL of chloroform solution containing 4 mg 9.0-nm size QDs and 6 mg 5.9-nm size MNPs was injected into 1 mL DTAB (dodecyltrimethylammonium bromide as a surfactant) aqueous solution (20 mg/ mL in Nanopure water), followed by thorough mixing. After removing the chloroform, the co-assembling process (Figure 3Aa) resulted in multifunctional multi-core particles of approximately 120 nm size consisting of a close-packed magnetoresponsive core and a fluorescent quantum dots shell (Figure 3Ab–d). An additional thermal annealing process results in supercrystalline core–shell-structured supraparticles. After functionalizing with polyethylene glycol (PEG), the supraparticles can be magnetically manipulated inside living cells while being optically tracked.

Polylactic-co-glycolic-acid (PLGA) hybrid nanostructures were synthesized by a double-emulsion (water-in-oil-in-water) technique, involving a hexane-based ferrofluid and PbS quantum dots (QDs) dispersed in toluene [145]. The ferrofluid mediated the encapsulation of magnetic and infrared emitting nanoparticles (PbS) in a polymeric matrix (Figure 3Ba–d) to provide magnetic–fluorescent imaging abilities to the resulting multi-core particles.

Oleic acid-coated PbS/CdS QDs and Fe_3_O_4_ NPs dispersed in chloroform were made water-dispersible by micellar encapsulation, which was due to hydrophobic van der Waals interactions between the hydrocarbon chains of oleic acid and DTAB used as surfactant [146]. The resulting self-assembled Fe_3_O_4_ and PbS/CdS supernanoparticles are aimed at synergistic dual-mode heating treatment for cancer therapy.

### 2.2. Induced Destabilization of a Ferrofluid

**Figure 4:**
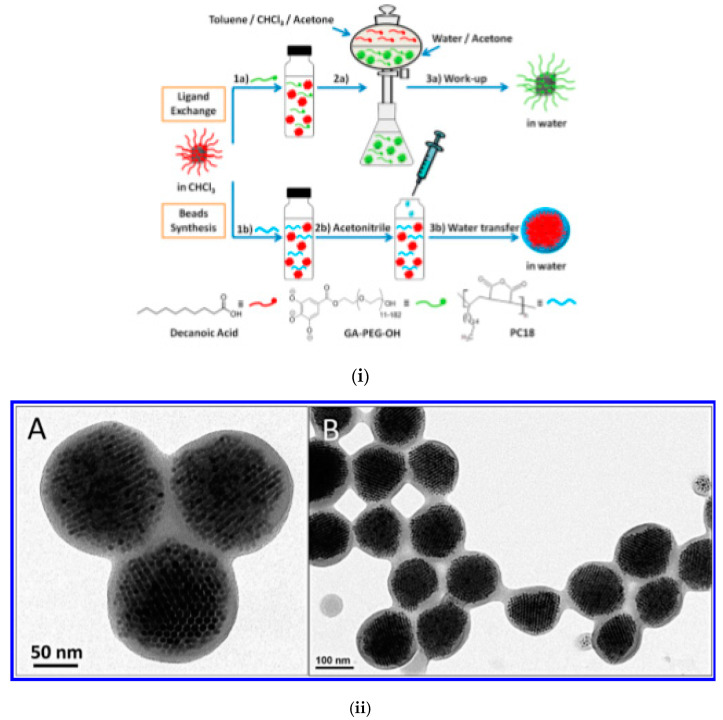

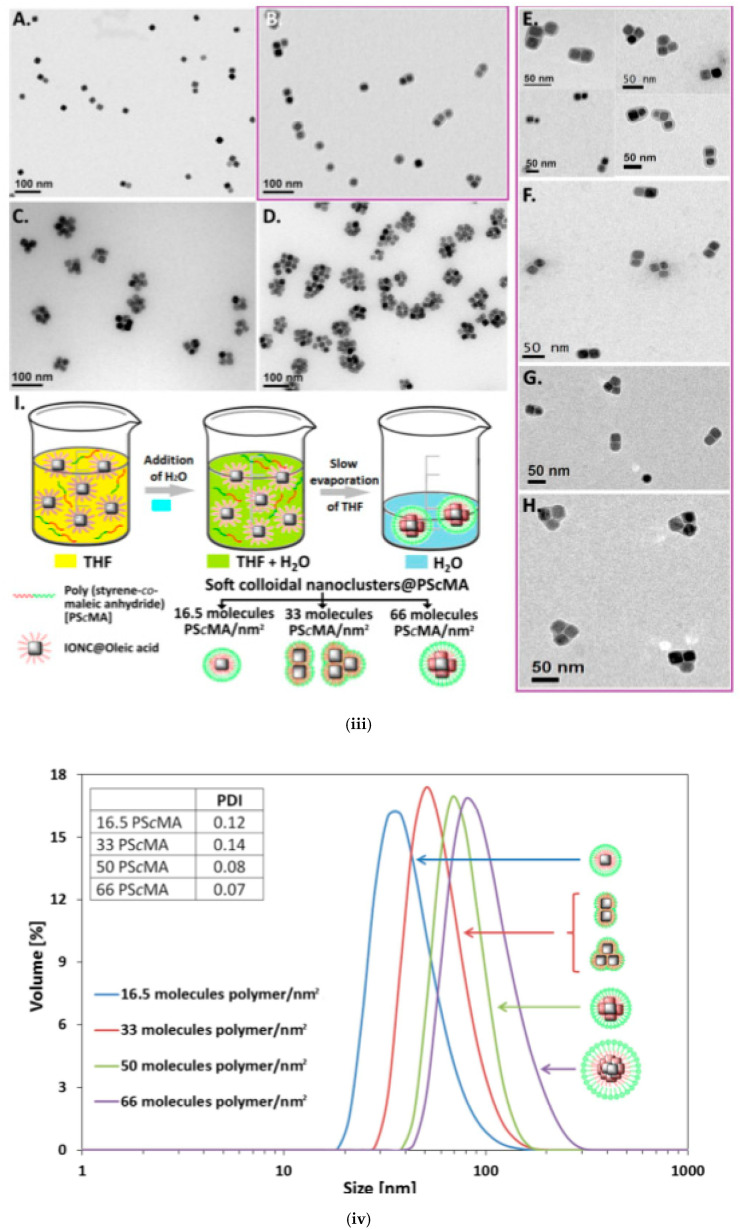
Controlled colloidal assembly of iron oxide nanoparticles from highly volatile ferrofluids. (**i**) (**a**) Starting from the same hydrophobic iron oxide nanocubes (IONCs) (23 ± 3 nm), IONCs are transferred in water by mixing the hydrophobic IONCs in toluene with the gallol-bearing PEG ligand (GA-PEG-OH) in the presence of a base (1a). The solution is shaken for a few seconds, and after acetone addition, the PEG-IONCs are extracted in water (2a). Finally, after organic solvent evaporation at reduced pressure, the PEG-IONCs solution is dialyzed to remove the excess of GA-PEG-OH (3a). This protocol provides the single-coated nanocubes in water. The MNBs instead are obtained by mixing the hydrophobic IONCs with a poly(maleic anhydride-alt-1-octadecene) polymer (PC18) in CHCl_3_ (1b). The solution is shaken for few seconds, and then, 1 mL of acetonitrile is added at a flow rate of 2 mL min−1 (2b). The MNBs are collected by magnetic sorting and redissolved in water (3b) (Reprinted with permission from [148]. Copyright 2015 American Chemical Society). (**ii**) Polymer encapsulated colloidal-ordered assemblies (COA). TEM images of polymer–COA at higher (**A**) and lower (**B**) resolution. The dark pattern (**A**) results from the ordering of the closed packed assemblies within the nanobeads, while the brighter gray ring is caused by the polymer shell (lower electron density) of around 20 nm thickness.) (Reprinted with permission from [65]. Copyright 2015 American Chemical Society). (**iii**) Scheme of the clustering protocol using 20 nm core–shell iron oxide nanocubes. Representative TEM micrographs of IONCs@PScMA in water and just after they have been prepared at a ratio of (**A**) 16.5, (**B**) 33, (**C**) 50 and (**D**) 66 polymer chains/nm^2^ of particle surface. (**E**–**H**) A collection of TEM images at higher magnification of dimers and trimers formed at the ratio of 33. Schematic representation of the formation of soft colloidal nanoclusters. (**iv**) Tuning the mean hydrodynamic diameter of clusters by different polymer amounts. Volume distribution of hydrodynamic size of soft colloidal clusters measured in water starting from 20 nm IONCs. The hydrodynamic diameter was adjusted between 38 and 99 nm. No aggregation of clusters was detected, as polydispersity index (PDI) values were between 0.07 and 0.14 (see inset) (reprinted with permission from [149]. Copyright 2017 American Chemical Society).

Highly ordered soft magnetic nanoclusters [147] were obtained by strongly polar solvent (acetonitrile) or amphiphilic polymer (poly(styrene-co-maleic anhydride) (PScMA)) induced the destabilization of a volatile (toluene, chloroform, or tetrahydrofuran-based) ferrofluid [148,149], Figure 4i–iv.

### 2.3. Magnetoliposomes

**Figure 5:**
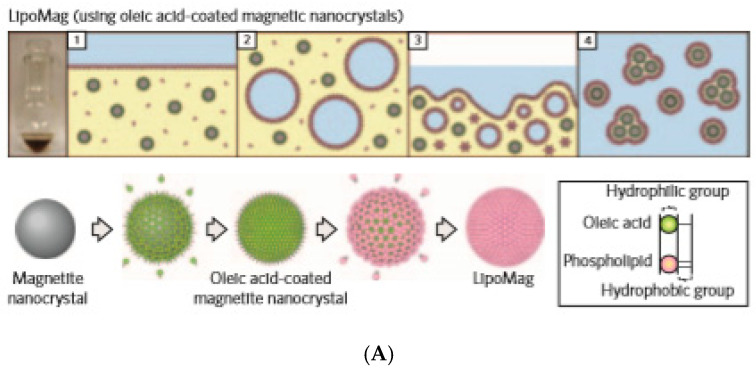

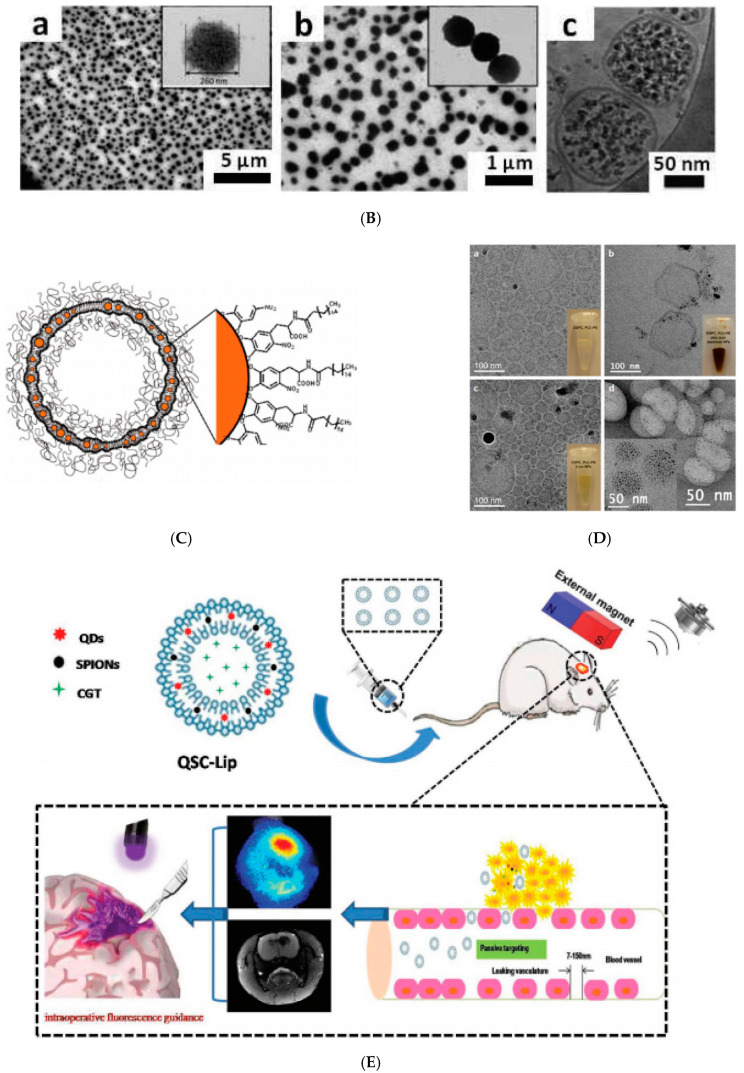
(**A**) Schematic showing the preparation (upper) and assembly (lower) of LipoMag. Oleic acid-coated magnetic nanocrystal cores and the lipid shells form through hydrophobic interactions (reprinted by permission from Copyright Clearance Center: Springer Nature, Nature Nanotechnology, [158], Copyright 2009). (**B**) (**a**,**b**) TEM and (**c**) cryo-TEM micrographs of UMLs prepared by an REV process. At low magnification, a large number of dense vesicles are observed with diameters 200 nm in average. MNPs are trapped inside unilamellar vesicles (**c**) and dipole−dipole interaction can occur as exemplified by magnification (**b**). (Reprinted with permission from [64]. Copyright 2012 American Chemical Society). (**C**) Schematic of liposomes containing iron oxide NPs in their bilayer. NitroDOPA–palmityl-stabilized iron oxide NPs are embedded in liposome membranes consisting of PEGylated and unmodified lipids; (**D**) Liposomes functionalized with iron oxide NPs. Cryo-TEM images of DSPC liposomes containing 5 mol % PEG(2)–PE that (**a**) were unmodified and incorporated (**b**) oleic acid-coated and (**c**) palmityl–nitroDOPA stabilized small iron oxide NPs. Insets show photographs of the respective PbS-based liposome dispersions where the lipid concentration was kept constant at 5 mg/mL. A comparison between (**a**) and (**c**) reveals no significant change of the spherical shape of liposomes upon loading their membranes with small, individually stabilized, iron oxide NPs. However, agglomerated, oleic acid-stabilized NPs seem to significantly distort the liposome shape. (**d**) TEM image of trehalose-fixed DSPC liposomes containing palmityl–nitroDOPA stabilized small NPs in their membranes. Liposomes were fixed with trehalose and air-dried on a carbon-supported Cu TEM grid where the carbon film had 3.5 μm diameter holes. While the large image was taken in a hole that was spanned by trehalose, the inset was imaged on the carbon support. Individually stabilized NPs with core diameters <5.5 nm are associated with liposomes. No NPs with core diameters >5.5 nm are seen. The inset indicates a high NP density of liposomes that were collapsed on the carbon support upon drying in air. (Reprinted with permission from Amstad et al. 2011a. Copyright 2011 American Chemical Society). (**E**) Liposome-integrated multiple-imaging agents and therapeutic drug for glioma-targeted delivery under exogenous magnetic field to accurately localize glioma. CGT, cilengitaide; QDs, quantum dots and SPIONs, superparamagnetic iron oxide nanoparticles were initially dispersed in chloroform. (Republished with permission of John Wiley and Sons, from [163]; permission conveyed through Copyright Clearance Center, Inc.).

Liposomes and, especially, magnetoresponsive liposomes are among the most promising vesicular drug carrier vehicles [150,151]. Allowing for encapsulation, retention, the membrane sealing off the interior of a hydrophilic volume from the environment, and the magnetic field-triggered release of the drug are the main attractive features of membranes-protected hollow nanocarriers for nanomedicine [152]. The loading of liposomes with iron oxide nanoparticles [153,154,155], mostly clusters preformed from aqueous [64,156,157] and organic [158,159] ferrofluids gives rise to magnetoliposomes with high magnetophoretic mobility and MRI contrast [160]. LipoMag composites [158] are assembled as oleic acid-coated magnetite nanocrystal cores with cationic lipid shells from a chloroform-based magnetic fluid through hydrophobic interactions [161,162] (Figure 5A).

Maghemite nanoparticles (9 or 7 nm) coated with citrate ligands and dispersed in water (Massart ferrofluid) or in a buffer were used for the preparation of Ultra Magnetic Liposomes (UMLs) encapsulating iron oxide nanoparticles in a volume fraction of up to 30% [64], Figure 5B. UMLs were prepared by a modified version of the reverse phase evaporation (REV) method [164]. This remarkable magnetic charge provides UMLs with high magnetic mobilities, MRI relaxivities, and heating capacities for magnetic hyperthermia [157]. Palmityl–nitroDOPA-stabilized iron oxide NPs were found to be spontaneously incorporated into liposome bilayers, whereas oleic acid-stabilized NPs agglomerated to form micelles [165], as seen in Figure 5C,D. The observed significant difference between palmityl–nitroDOPA and oleic acid-stabilized NPs can be related to the irreversible binding of nitroDOPA versus the reversible adsorbing of oleic acid to the iron oxide surfaces, which favors the agglomeration of OA-coated NPs. Hydrophobic SPIONs and QDs initially dispersed in chloroform were co-encapsulated inside a lipid membrane to provide “all in one” nanocarriers–theranostics liposomes for glioma targeting [163], as shown in Figure 5E.

**Figure 6:**
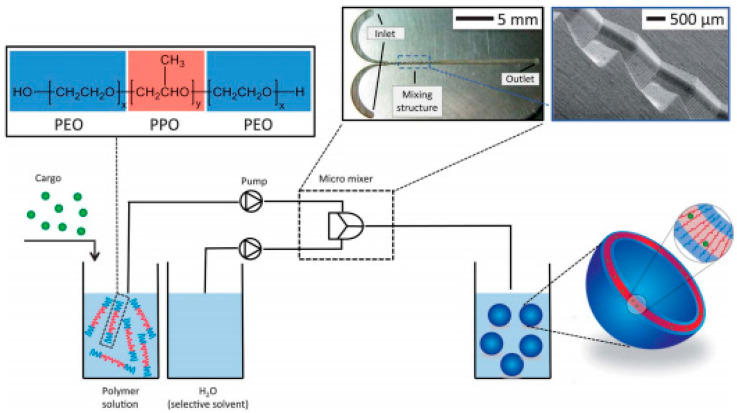
Continuous preparation of loaded magnetic polymersomes using a tetrahydrofuran-based ferrofluid: the starting polymer solution (PEO–PPO–PEO in tetrahydrofuran) is diluted with water, the selective solvent for the PEO block, and induces polymersome self-assembly. The microstructured mixing device is a stainless steel caterpillar micromixer with twelve mixing steps and a mixing channel with an inner volume of 10 mL. Hydrophobic agents were loaded in situ by simply adding the cargo (magnetic nanoparticles or drug molecules) to the starting polymer solution prior to mixing. Due to the hydrophobicity of those compounds, incorporation in the hydrophobic part of the vesicle membrane occurs. Prior carboxylation of the end-groups of the polymer enables further surface functionalization and conjugation to specific targeting moieties. (Republished with permission of John Wiley and Sons, from [166]; permission conveyed through Copyright Clearance Center, Inc.).

The encapsulation of hydrophobically coated IONPs from a tetrahydrofuran-based ferrofluid together with camptothecin anticancer drug into a PPO block of Pluronic vesicles (Pluronic L121 (PEO–PPO–PEO, as purchased or carboxylated by succinic anhydride)) provided a scalable continuous manufacturing procedure to obtain multi-core theranostic drug delivery vehicles [166], as schematically represented in Figure 6.

### 2.4. Co-Assembling in Aqueous Solution

**Figure 7:**
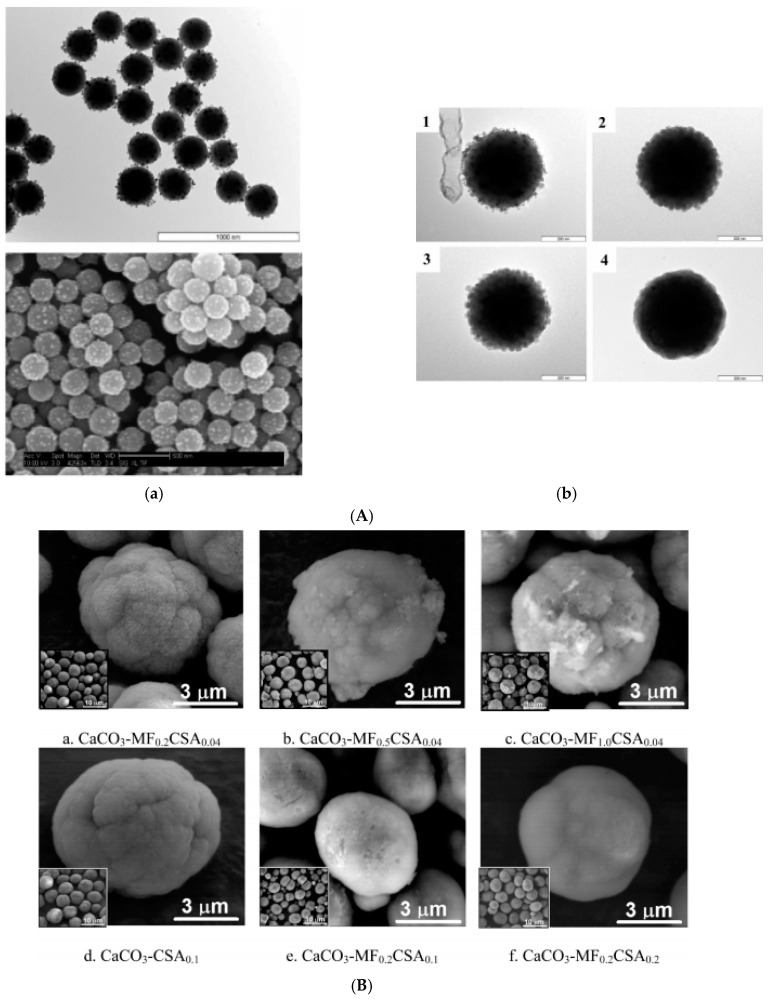
(**A**) (**a**) TEM (top) and corresponding SEM picture (bottom) of silica–cobalt ferrite nanocomposite particles (**b**) Silica growth onto composite particles monitored by TEM, which shows the gradually increasing silica layer thickness from no silica present in picture 1 to a fully grown silica layer in picture 4. (Reprinted with permission from [52] Copyright 2005 American Chemical Society). (**B**) Magnetoresponsive chondroitin sulfate-functionalized CaCO_3_ microparticles. The influence of CSA and magnetic fluid (MF) content on the CaCO_3_−MF−CSA composite size, shape, and morphology, as evidenced by SEM (reprinted with permission from [167]. Copyright 2013 American Chemical Society).

Magnetic nanoparticles attached to a silica core with variable size provide a composite particle with a tunable-induced magnetic moment. Moreover, by applying an external silica coating, the thickness of the shell allows tuning also the dipolar interactions between particles at contact. The preparation procedure of these composites developed in [52] uses aqueous ferrofluid with maghemite or cobalt ferrite nanoparticles and an aqueous dispersion of silane-coupling agent-coated silica particles, ensuring the chemical attachment of magnetic nanoparticles to the silica core. Depending on the core size and shell thickness, the overall size of these superparamagnetic composite particles is between 150 and 200 nm (Figure 7A).

Magnetoresponsive chondroitin sulfate-functionalized CaCO_3_ microparticles (Figure 7B) were obtained by crystallization from supersaturated aqueous solutions in the presence of oleic acid-stabilized magnetite nanoparticles as a water-based magnetic fluid and a natural strong–weak polyanion, chondroitin sulfate A (CSA) [167]. Accordingly, the growth mechanism of superparamagnetic microparticles involves the chains of CSA and the surfactant-coated magnetite nanoparticles, which could electrostatically accumulate a large amount of Ca^2+^ and carbonate ions; Ca^2+^ ions form an ionically cross-linked network with the carboxylate groups on the CSA and oleic acid. The microparticle characteristics investigated by physicochemical methods (SEM, TEM, X-ray diffraction, Raman spectroscopy, flow particle image analysis, particle charge density, and electrokinetic measurements) depend on the initial MF amount and polymer concentration. Biocompatibility and also enhanced pH stability make their use in bio-related applications attractive.

## 3. Non-Spherical Multi-Core Superparamagnetic Assemblies

Superparamagnetic, multi-core nanocomposites built up by nanometer-sized magnetic nanoparticles exhibiting anisotropic morphologies [168] are highly interesting in the biomedical field as drug delivery systems, magnetic hyperthermia mediators, biosensors, MRI contrast agents, and bioseparators, due to their anisotropic magnetic response, high surface area, high magnetic moment, and high magnetic mobility [169].

Different synthetic approaches have been developed so far for the generation of anisotropically shaped superparamagnetic supraparticles starting from ferrofluids, including evaporation-guided, emulsion-templated and magnetic field-assisted self-assembly, supramolecular polymerization, electrospinning, ink-jet printing, and lithography-based approaches. In the following sub-sections, selected literature examples related to the aforementioned fabrication routes are provided and briefly discussed.

### 3.1. Evaporation-Guided Self-Assembly

The evaporation-guided self-assembly of colloidal ferrofluids has been employed by several research groups for obtaining superparamagnetic anisotropic supraparticles of various shapes and with sizes ranging from several µm up to mm.

In this synthetic route, a liquid-repellent surface is used, on which ferrofluid droplets are left to dry in a controlled manner, resulting in the formation of superparamagnetic multi-core supraparticles [170,171].

**Figure 8:**
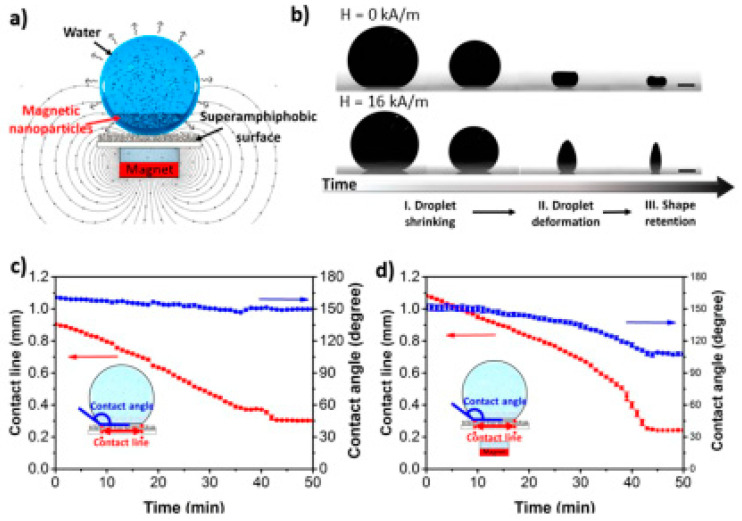
Formation of large 3D superparamagnetic structures by making use of the magnetic properties of ferrofluids. (**a**) Experimental system used for the production of supraparticles by evaporation-guided assembly of a magnetic NPs dispersion on a superamphiphobic surface. (**b**) Evolution of a 3 wt % droplet during drying without (upper panel) and with (bottom panel) magnetic field. Scale bars are 0.5 mm. Drying curve of the droplet (**c**) without and (**d**) with magnetic field. Insets represent the dimensions measured during drying (reprinted with permission from [172]. Copyright 2019 American Chemical Society).

Hu and co-workers described the preparation of superparamagnetic supraparticles having distinct anisotropic shapes, starting from a magnetic colloidal aqueous suspension consisting of hybrid Fe_3_O_4_/polystyrene nanoparticles, stabilized with sodium dodecyl sulfate (SDS) [172]. The ferrofluid was left to dry on a superamphiphobic surface [173] in the presence of an externally applied magnetic field, resulting in the entrapment of the transient suspension droplet shapes upon evaporation. The authors demonstrated that different 3D anisotropic morphologies can be obtained including cones, barrels, and two towers (Figure 8) upon altering the magnetic orientation, strength, and initial nanoparticle concentration. Moreover, since the presented approach is based on the evaporation-induced self-assembly starting from ferrofluids, it allows for the assembly of co-suspensions consisting of different NP types (such as TiO_2_), thus providing anisotropic binary supraparticles.

In another example, evaporation-induced self-assembly applied on Fe_3_O_4_/SiO_2_ core–shell nanoparticle aqueous dispersions deposited on curved, superhydrophobic surfaces was conducted to the formation of ellipsoidal anisometric magnetic Janus supraparticles [174]. The latter was accomplished in the presence of a magnetic field, guiding the accumulation of the magnetic nanoparticles at specific locations within the supraparticles.

Magnetic halloysite nanotubes (HNT), promising nanocomposites for MRI or magnetic hyperthermia, were prepared by loading preformed oleic acid-coated superparamagnetic magnetite nanoparticles (physical size ≈6 nm; hydrodynamic diameter of ≈10 nm) into HNT [175]. The OA-stabilized MNPs of a hexane-based ferrofluid were selectively loaded on the tetradecylphosphonic acid (TDP)-modified inner lumen of halloysite nanotubes in a slow evaporation process exploiting vacuum–N_2_ cycles.

O’Mahony and co-workers prepared superparamagnetic microparticles of various morphologies, including dimpled and crumpled microparticles, by means of emulsion templated self-assembly, using oil-in-water emulsions [125]. Ferrofluids consisting of hydrophobic oleic acid-coated Fe_3_O_4_ NPs were employed for this purpose. The authors demonstrated among others that the ferrofluid concentration and the density of the oleic acid chains covering the nanoparticle surfaces significantly affect the morphology of the resulting microparticles. Such systems, exhibiting high magnetic mobilities, high surface areas, and consequently high binding affinities, are very promising in magnetic bioseparation processes.

In a final example, 2D and 3D mesocrystalline films were generated from colloidal dispersions of oleic acid-stabilized magnetite nanocubes in toluene via self-assembly under slow evaporation conditions [176]. The self-assembly process is driven by the NP dipolar magnetic attractive forces and the presence of an external magnetic field. In the case of the 2D mesocrystalline films, the generation of two distinct Fe_3_O_4_ nanocube superstructures was observed, having the same orientational order and *p* 4 mm and *c* 2 mm layer symmetries, while slightly distorted fcc superlattices were found in the case of the 3D mesocrystalline films.

### 3.2. Magnetic Field-Assisted Self-Assembly

**Figure 9:**
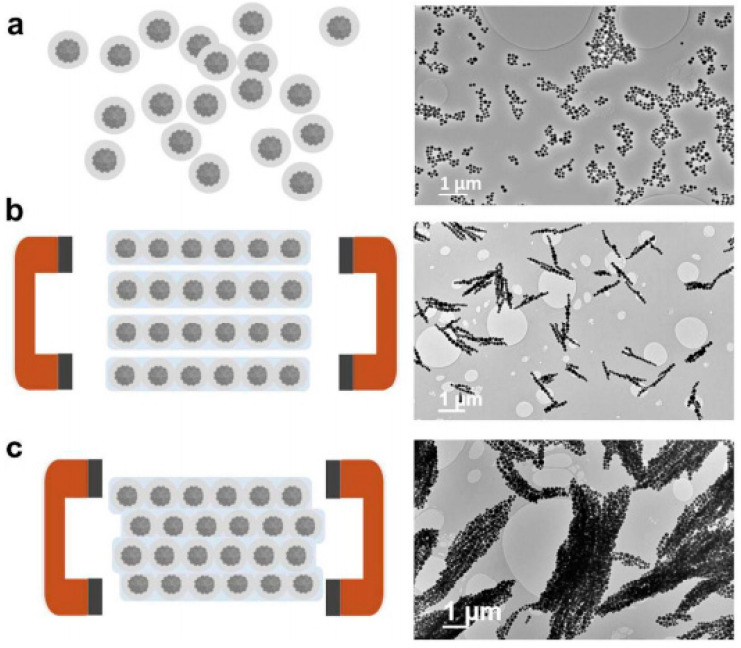
Schematic representation of the fabrication process (left-hand image) and the corresponding transmission electron microscopy images (right-hand image) of (**a**) silica-coated superparamagnetic nanoparticle clusters, (**b**) nanochains, and (**c**) nanobundles (reprinted with permission from [177]. Copyright 2015 American Chemical Society).

Highly stable aqueous ferrofluids consisting of superparamagnetic maghemite (γ-Fe_2_O_3_) nanoparticle clusters encapsulated within silica shells of controllable thicknesses were synthesized and further used in the generation of 1D, highly anisotropic nanostructures. These included nanochains and nanobundles (Figure 9) formed in the presence of a magnetic field, which was due to the development of magnetic dipole–dipole interactions [177]. The incorporation of polyvinylpyrrolidone (PVP) in the aqueous superparamagnetic NP cluster suspensions provided stability to the assembled 1D structure that was irreversibly “locked” into fixed nanochain/nanobundle morphologies by incorporating an additional silica layer via hydrolysis/condensation reactions.

**Figure 10:**
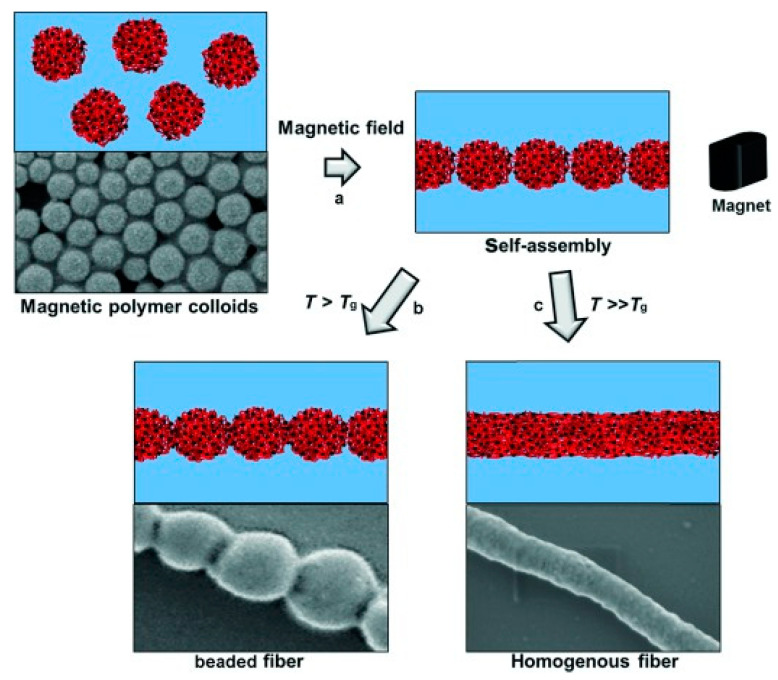
Magnetic self-assembly (**a**) and fusion (**b**,**c**) of magnetic polymer colloids in water.

Bannwarth et al. described the construction of nanofibers via the magnetic-field assisted self-assembly of spherical monodisperse and polydisperse magnetic nanoparticles dispersed in aqueous media (ferrofluid) [178,179,180]. Polystyrene nanoparticles with embedded oleate-capped magnetite nanoparticles were prepared by miniemulsion polymerization. Their high magnetic content ensured strong interparticle magnetic attraction in the presence of a magnetic field, thus giving rise to nanofibrous assemblies [178]. Among others, it was demonstrated that the degree of fusion of the nanoparticles and consequently the nanofiber morphology could be tuned by controlling the temperature of the aqueous solution. More precisely, when the solution temperature exceeded the glass transition temperature (T_g_), a high degree of nanoparticle fusion occurred, giving uniform nanofibers, while at solution temperatures below the Tg, necklace-like morphologies were obtained due to partial nanoparticle fusion (Figure 10). By employing the same fabrication strategy starting from polystyrene-capped colloidal magnetite nanoparticles exhibiting a Janus morphology [181], anisotropic zig-zag nanomorphologies were generated in the presence of a magnetic field.

Increasing the temperature above the glass transition temperature (T_g_) provides enough polymer chain flexibility and leads to a linear sintering process. Temperatures close to the T_g_ yield a low degree of fusion and a necklace-like morphology (b). For temperatures well above the T_g_, a larger degree of fusion is observed, and completely merged particles form a homogeneous fiber containing homogeneously distributed iron oxide nanoparticles (c) (republished with permission of John Wiley and Sons, from [178]; permission conveyed through Copyright Clearance Center, Inc.).

**Figure 11:**
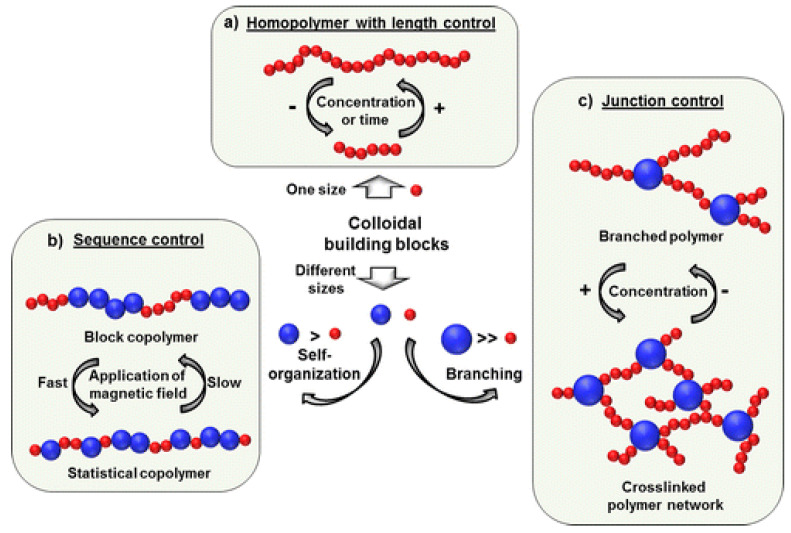
Controlled assembly and fusion of superparamagnetic polystyrene nanoparticles; schematic representation. (**a**) Insertion of a monodisperse nanoparticle dispersion yields predominantly linear nanochains. Depending on the concentration of the nanoparticle dispersion (and the growth time), longer or shorter chains can be obtained. (**b**) In the case of a polydisperse sample, the different sized particles (colored in red and blue representing small and large particles, respectively) can self-organize into blocks of larger and smaller particles, resulting in colloidal block copolymers or in rather statistical fashion. A slow increase of the external magnetic field assembles the particles in a rather block-like pattern, while a fast increase assembles the particles in a rather statistical fashion. (**c**) The insertion of particles with even larger size differences enables the introduction of junction points. Here, more than two small nanoparticles assemble around a large particle creating a junction point within the nanoparticle chain. By increasing the concentrations of nanoparticles in the dispersion, networks of cross-linked chains can be obtained. (Reprinted with permission from [179]. Copyright 2015 American Chemical Society).

In the case of the polydisperse magnetic nanoparticle dispersions, the generation of different anisotropic morphologies with controlled lengths and architectures could be obtained, including linear nanochains of controllable lengths, architectures resembling block and statistical copolymers generated by tuning the external magnetic field, as well as branched and crosslinked network architectures (Figure 11) [179].

Water-dispersible magnetic iron oxide nanorings (approximately 133 nm overall hydrodynamic size) with a unique ferrimagnetic vortex-domain structure, in which magnetization is circumferential to the ring without stray fields, have a much higher saturation magnetization and large hysteresis loop in comparison with SPIONs, reduced dipole–dipole interactions, and good colloidal stability, providing a highly efficient hyperthermia agent [182].

Functionalized Janus magnetic nanoparticles were synthesized by following the grafting approach [183]. More precisely, the selective grafting of either polystyrene sodium sulfonate or polydimethylamino ethyl methacrylate occurred on the exposed surfaces of poly (acrylic acid)-functionalized magnetite nanoparticles anchored on silica beads. Upon formation, these nanoparticles were detached from the silica surfaces and further used as building blocks for the pH-triggered reversible formation of small, elongated anisotropic clusters. The experimental results were found to be in line with Monte Carlo simulations performed using a modified Monte Carlo cluster–cluster aggregation algorithm.

Anisotropic microrod supraparticles have been synthesized by means of magnetic field-assisted self-assembly [184,185]. In one such example, microrod supraparticles generated via the magnetic field-assisted assembly using ferrofluids, composed of superparamagnetic iron oxide nanoparticles (≈10 nm), were studied by Magnetic Particle Spectroscopy (MPS) [185]. The presence of a magnetic field during the formation of such supraparticles is essential for obtaining anisotropic morphologies, while in the absence of a magnetic field, isotropic supraparticles are formed. The MPS signals of the isotropic assemblies were similar to those corresponding to the individual nanoparticles, while in the case of the anisotropic assemblies, significant MPS signal enhancement was recorded. Based on the above, MPS has proven to be a valuable method that can be used to distinguish isotropic from anisotropic magnetic supraparticle assemblies.

### 3.3. Magnetic Nanoparticle Assemblies on Surfaces

Commercially available, monodispersed, single-domain spherical magnetic nanoparticles stabilized in aqueous media (ferrofluids) were used at low solution concentrations (below 1% vol) in the construction of 3D NP assemblies on silicon surfaces [186,187]. Neutron reflectivity studies were performed to investigate the self-assembly process. The NPs were stabilized in aqueous solutions in the presence of oleic acid and a carboxylic acid-functionalized polymer, with the latter enabling the development of electrostatic attractive forces with the amino-functionalized silicon surface. Experimental findings suggested the formation of a close-packed NP monolayer directly deposited on the silicon surface followed by the deposition of additional NP layers, resulting in the generation of 3D multilayer NP arrays of various thicknesses and densities, depending on the strength of the applied magnetic field. In those 3D NP arrays comprised of both closely packed and loosely packed NP layers, both relaxation mechanisms (Néel and Brownian) [188] were considered for the interpretation of their magnetic behavior.

### 3.4. Electrospinning 

Electrospinning is considered to be one of the most versatile methods that is used in the fabrication of sub-nano, nano-, and microfibers [189,190]. The electrospinning technique is simple, cost-effective, and industrially scalable [191,192], providing a straightforward way to produce long and continuous polymer fibers by using electrical forces [193,194]. Moreover, electrospinning enables the incorporation of inorganic nanoparticulates toward the production of fibrous nanocomposites. Furthermore, the possibility provided by this method for co-processing mixtures of different polymers and of polymers with small organic molecules generates new pathways for altering the chemical composition of the fibers and therefore expanding and tuning their properties including mechanical strength, physical and thermal properties, porosity, wettability, and permeability.

**Figure 12:**
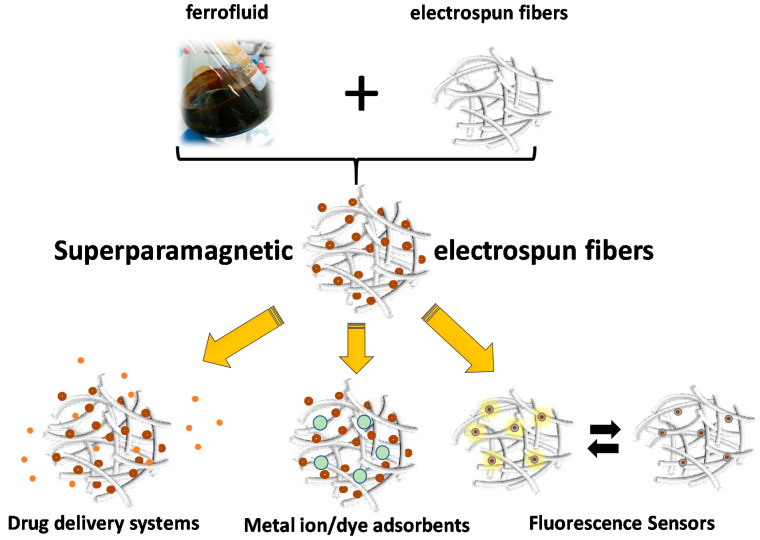
Superparamagnetic electrospun fibers generated from ferrofluids and electrospun nano-and microfibers.

In recent years, the fabrication, characterization, and applications of superparamagnetic polymer-based electrospun fibers has been made using highly stabilized superparamagnetic ferrofluids and polymers of various chemical compositions and functionalities (Figure 12). These include the preparation of white, superparamagnetic paper consisting of electrospun cellulose microfibers doped with iron oxide nanoparticles [195], superparamagnetic electrospun fibrous membranes consisting of *β*-ketoester-functionalized methacrylate-based polymers, and preformed, oleic acid-coated Fe_3_O_4_ nanoparticles that were further evaluated as adsorbents for Eu(III) from aqueous media [196,197], surface-modified magnetic polyvinylpyrrolidone/chitosan blended electrospun nanofibers that were investigated as carriers in cell and enzyme immobilization [198], electrospun polymer–Fe_3_O_4_ nanocomposite mats studied as dye adsorbents [199], and superparamagnetic electrospun nanocomposite fibers designed for use in the biomedical field [200,201]. Moreover, core–shell γ-Fe_2_O_3_/SiO_2_ NPs, functionalized with fluorescent rhodamine B molecules, were combined with cellulose acetate electrospun fibers to yield multifunctional fluorescent fibrous nanocomposites employed as ammonia gas and pH sensors [202].

However, in all these examples, the magnetic nanoparticles were homogeneously dispersed within or onto the surfaces of the electrospun fibers as single domain NPs, and no supraparticle assemblies were generated during electrospinning or upon post-magnetization of the as-prepared fibrous mats.

Nevertheless, there are a few literature examples demonstrating the applicability of electrospinning in the generation of anisotropic multi-core nanoparticle assemblies. In one such example, silica-coated magnetite core–shell NPs spontaneously self-assembled into multi-core assemblies using electrospinning followed by treatment in aqueous ethanol solution under basic pH conditions [203]. More precisely, electrospinning was used to fabricate fibers with variable magnetic content, consisting of polyvinylpyrrolidone, Fe_3_O_4_ NPs, tetraethylorthosilicate (TEOS), and cetyltrimethylammonium bromide (CTAB) that were subsequently immersed in an aqueous ethanol solution at pH 9.0. The latter led to the formation of spherical, core–shell Fe_3_O_4_/SiO_2_ NPs that spontaneously self-assembled, creating supraparticle assemblies exhibiting high magnetization and superparamagnetic properties. According to the authors, the role of the electrospun fibers in the NP assembly process is important, since the incorporation of the different chemical substances within the nanofibrous templates promotes the development of specific interactions between them, thus facilitating the self-assembly process within a constrained nanoenvironment.

Bannwarth et al. reported the formation of ellipsoidal nanoclusters, starting from an octane-based ferrofluid consisting of iron oxide NPs by means of emulsion electrospinning, which is based on an aqueous emulsion containing Fe_3_O_4_ NP-loaded octane droplets [180]. The produced nanoclusters embedded within poly(vinyl alcohol) (PVA) electrospun fibers exhibited superparamagnetic properties and high saturation magnetization. By dissolving the hydrophilic PVA fibers in water accommodating the ellipsoids, the latter can be easily isolated in the form of aqueous dispersions, thus enabling their further exploitation in biomedical applications.

By applying a magnetic field perpendicular to the electric field used during electrospinning, spherical, superparamagnetic nanoparticles aligned in 1D arrays within e-polycaprolactone microfibers can be obtained [204]. The length of these magnetic arrays varied, depending on the strength and uniformity of the applied magnetic field. Concerning magnetic behavior, despite nanoparticle alignment, the observed magnetic properties resemble those of individual NPs rather than those of an interconnected nanowire assembly.

### 3.5. Supramolecular Approaches

Supramolecular assemblies consist of building units of organic or inorganic nature, held together by non-covalent, supramolecular interactions including ionic, hydrophobic, van der Waals, hydrogen, and coordination bonds [205]. Supramolecular synthetic methods that are based on non-covalent metal–ligand interactions developed between organic and inorganic components have been exploited in the fabrication of organic–inorganic hybrid materials [206]. Among supramolecular structures, chiral-engineered supraparticles are of utmost importance to achieve the better control of drug delivery systems and other nanomedicine-related applications [207].

**Figure 13:**
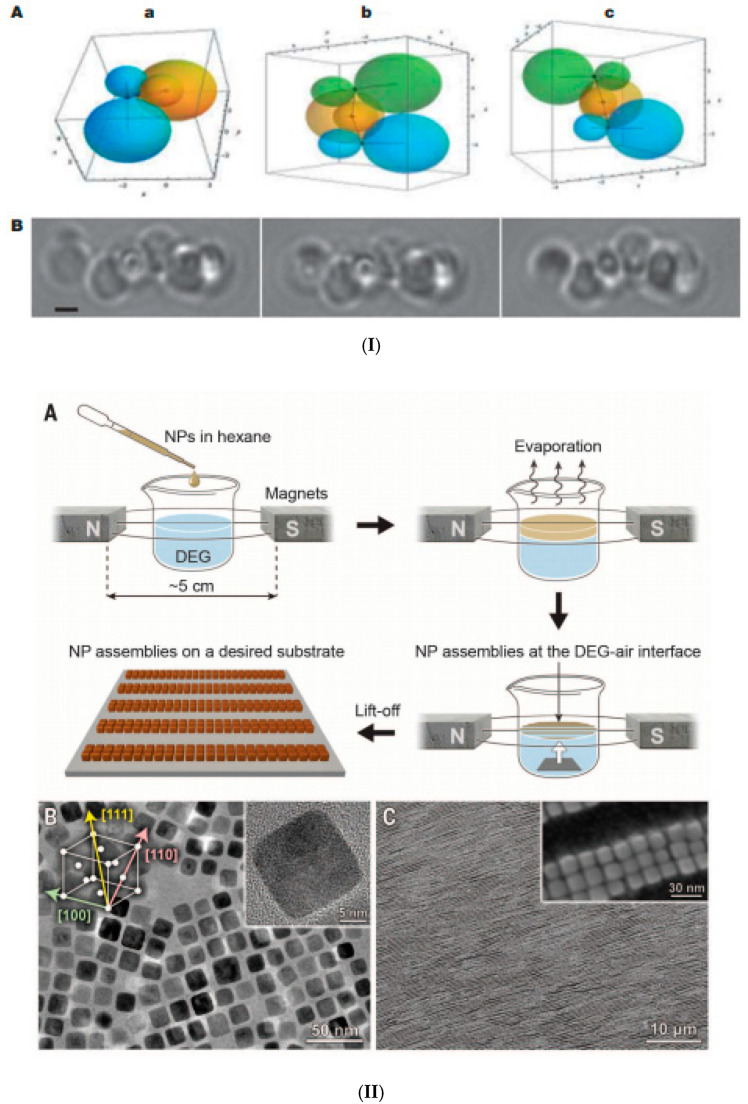
(**I**) Magnetoresponsive dumbbell assembly. (**A**) Schematics showing a pair of dumbbells (**a**) and the two ways in which a third dumbbell could be added to that pair (**b**,**c**). (**B**) Optical microscopic images showing the kinetic pathway of a dumbbell that diffuses and assembles onto a five-dumbbell helix. Scale bar, 1 mm. (Reprinted by permission from Copyright Clearance Center: Springer Nature, Nature, [208], Copyright 2015). (**II**) Self-assembly of one-dimensional nanocube belts. (**A**) Schematic representation of the experimental setup. (**B**) Low- and high-magnification transmission electron microscopy (TEM) images of the building blocks, ≈13-nm Fe_3_O_4_ nanocubes. The (111), (110), and (100) crystallographic directions correspond to the easy, intermediate, and hard axes of magnetization, respectively. (**C**) Low- and high-magnification scanning electron microscopy (SEM) images of belts_100_ [209]. Reprinted with permission from AAAS).

Helical magneto-responsive superstructures were obtained in magnetic field-directed self-assembly procedures using ferrofluids. Hydrophobic micrometer-sized silica particles dispersed in an octane-based ferrofluid take part in droplets formed in a careful emulsion procedure providing, after solvent evaporation, the dumbbell-type configuration presented in Figure 13I [208]. The helical structure develops as shown schematically in Figure 13IAa,b,c, as a result of an increasing magnetic interaction plus steric repulsion, for certain values of the ratio of the spheres. The movie frames in Figure 13IB illustrate the process starting from a single dumbbell to the final helical structure.

Singh et al. reported the formation of helical superstructures promoted by the self-assembly of surfactant (oleic acid)-stabilized Fe_3_O_4_ nanocrystals of a hexane-based magnetic colloid (ferrofluid) into various anisotropic shapes including cubes, rounded cubes, octahedra as well as heterodimeric Ag–Fe_3_O_4_ nanoparticles [209].

The assembly process (Figure 13IIA) involved the deposition of the surfactant-stabilized nanocrystals (Figure 13IIB) at the diethylene glycol (DEG)–air interface, which was followed by the application of an external magnetic field of variable strength that led to their alignment. Upon solvent (hexane) evaporation, helical nanocrystal superstructures were generated including single-, double-, and triple-stranded helices (Figure 13IIC).

**Figure 14:**
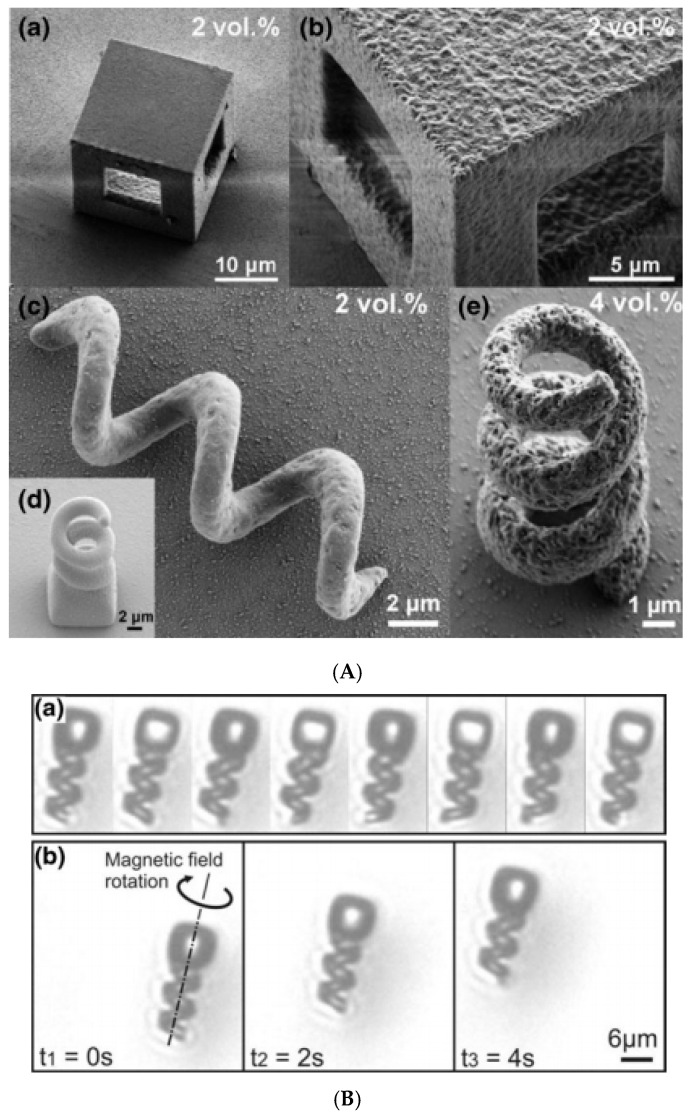
Swimming magnetic microrobots. Ferrofluid used for preparation: 11 nm magnetite NPs in γ-butyrolacton (GBL) carrier liquid. (**A**) SEM images from magnetic polymer composite (MPC) hollow cube (20 × 20 × 20 μm with 2 μm wall thickness and four 10 × 10 μm windows) with 2 vol.% Fe_3_O_4_ nanoparticle concentration (**a**). Closer view of the cube (**b**). Helical microstructure with 2 vol.% Fe_3_O_4_ nanoparticle concentrations (**c**) and with a cube-like base for better fixation to the substrate (**d**). Helical microstructure with 4 vol.% (**e**). (**B**) Swim test in water of MPC helical structures with 2 vol.% Fe_3_O_4_ superparamagnetic nanoparticle concentration. (**a**) Microscope image sequence showing a full rotation of the helical structure around its helical axis. (**b**) Microscope image sequence showing propulsion of the helical structure. The magnetic field strength and input frequency were set to 8 mT and 4 Hz, respectively. A distance of approximately 12 μm (forward plus drift motion) was covered in 4 s. (reprinted by permission from Copyright Clearance Center: Springer Nature, Biomedical Microdevices, [212], Copyright 2013).

Micro-and nanomotors involving helical structures are very promising tiny devices designed to fulfill various tasks in biology and medicine [210]. Inspired by bacterial flagellum propulsion, rotating magnetic field-driven helical motors proved to be more efficient compared to engines pulled with field gradients, especially when the size of the device decreases or when the source of the magnetic field is at a distance. Swimming superparamagnetic microrobots were manufactured using an organic solvent (γ-butyrolactone (GBL))-based ferrofluid with 11-nm diameter magnetite nanoparticles and a photocurable epoxy biocompatible polymer (SU-8) [211,212], as shown in Figure 14A,B.

### 3.6. Ink-Jet Printing and Lithography-Based Approaches

Ink-jet printing is a non-contact technique that enables the direct deposition of complex patterns on various surfaces, making use of very small volumes of solutions or suspensions. During the last years, this technology has attracted considerable attention in the field of organic–inorganic hybrid materials, enabling the precise deposition and patterning of polymer/nanoparticle inks onto selected surfaces [213].

Ink-jet printing using ferrofluids with monodisperse superparamagnetic poly(4-styrenesulfonic acid-*co*-maleic acid) sodium salt-protected Fe_3_O_4_ particles dispersed in H_2_O/ethylene glycol mixtures combined with magnetic guiding has been introduced by Gao et al. to fabricate 1D single particle arrays with controlled length and highly anisotropic magnetization [214]. Such 1D magnetic arrays could be exploited in different bio-related applications [94,215].

Tavacoli et al. presented a nice synthetic lithography-based approach involving the self-assembly of 300 nm mean size silica-coated superparamagnetic nanoparticles of a colloidal suspension into magnetic sub-5 micron arbitrary-shaped prisms or cylinders having high (50 wt %) magnetic content. More precisely, silica-coated superparamagnetic colloidal nanoparticles were introduced in ethoxylated trimethylolpropanetriacrylate employed as the monomer, and the mixture was placed into micron-sized PDMS wells. Upon UV irradiation, polymerization occurred, resulting in the formation of monodispersed micromorphologies of various sizes and shapes governed by the geometrical characteristics of the PDMS molds [216]. The applied methodology allows for a close packing of the superparamagnetic colloids, thus resulting in high magnetic susceptibilities. Moreover, by applying a magnetic field, the particles self-assembled into anisotropic chains, including necklace structures and rods.

Ferrofluid-based synthesis procedures and characteristic sizes of spherical and various non-spherical shape multi-core nanoparticles designed for biomedical applications are summarized in Table 1.

**Table 1:** Spherical and non-spherical multi-core magnetic particles.

Multi-Core Magnetic Particles	Primary Single-Core IONP	Preparation Method	Mean Size (nm)	M_sat_ (emu/g)	References
	**Spherical particles**				
MCIO/CMD	-	modified alkaline precipitation method	40–80 ^a^	-	[63,84]
MCIO/CTAB; MCIO/PEI; MCIO/PAA	Fe_3_O_4_/OA-Oam in hexane	emulsion	30–88 ^a^	60	[122]
MCIO/SDS	IONP/fatty acid (commercial product)	emulsion	40–200 ^a^	62	[123]
MCIO/SDS/hydrogel poly(NIPAM-AA)	IONP/fatty acid (commercial product)	emulsion/precipitation polymerization	64 ^a^; 80 ^b^	-	[124]
MCIO/PEG-AA	Fe_3_O_4_/OA in hexane(ferrofluid)	emulsion-templated	430–660 ^a^	7.5–24.8	[120]
MCIO/PEI/PAAMA	Fe_3_O_4_/OA in hexane (ferrofluid)	emulsion	≈1200 ^a^	-	[125]
MCIO/SDS-Tween 85; MCIO/CTAB-Tween 85; MCIO/Pluronic PE 6800	Fe_3_O_4_/OA in octane (ferrofluid)	emulsion	50–300 ^a^	57	[93]
MCIO/PBMA-g-C12	MnFe_2_O_4_/OA	emulsion	80 ^a^	11–32	[182]
MCIO in soybean, corn, cottonseed, olive oil or MCT/PEG-DSPE	Fe_3_O_4_/OA in toluene (ferrofluid)	emulsion	30–95 ^b^	-	[217,218]
MCIO/SDS/PAA MCIO/SDS/PNIPAM-PAA	Fe_3_O_4_/OA Toluene ferrofluid	emulsion/radical polymerization	100–200 ^a^	43–46.8	[127]
Silica-coated magnetic nanocapsules (SiMNCs)	Fe_3_O_4_/OA in octane ferrofluid	emulsion/silica coated of Fe_3_O_4_-polystyrene nanospheres/polystyrene burned	100 ^a^	45	[219]
MCIO/Protoporphyrin IX;	Hydrophobic SPIONs in toluene	emulsion	37 ^b^	-	[130]
MCIO/chlorin e6	Fe_3_O_4_/OA in toluene (ferrofluid)	emulsion	96.38 ± 4.6 ^b^	-	[135]
magnetoresponsive supraparticles (SPs)	CoFe_2_O_4_/OA in cyclohexane (ferrofluid)	emulsion	105–734 ^a^	-	[138]
SPs/DTAB/CdSe-CdS (QDs)	Fe_3_O_4_ in chloroform ferrofluid	emulsion	120 ^a^	15.2	[144]
MCIO/PLGA/ PbS(QDs)	Fe_3_O_4_/OA in hexane (ferrofluid)	double emulsion	100 nm–1 µm ^a^	55	[145]
MCIO/PS-*b*-PAA/pyrene/PVA	Hydrophobic SPIONs in chloroform	emulsion	180 ^a^	-	[141]
MCIO/GA-PEG-OH	Hydrophobic IONP (ferrofluid)	induced destabilization of ferrofluid	173 ^b^	60	[148]
MCIO/PScMA	IONP/OA in THF (ferrofluid)	induced destabilization of ferrofluid	38–99 ^b^	314–407 emu/cm^3^	[149]
Magnetic liposomes	IONP/OA in chloroform (ferrofluid)	coating of IONP with lipid shells	115–401 ^a^	-	[158]
MCIO/Polymer vesicle (Pluronic)	IONP/OA dispersed in tetrahydro furane (ferrofluid)	IONP embedded in polymer vesicle by microfluidic mixing	≈160 ^a^	-	[166]
MCIO/liposomes	ã-Fe_3_O_4_/citrate in water (ferrofluid)	encapsulation of IONP into the liposomes	200 ^a^	3 × 10^5^ A/m	[64]
MCIO/liposomes-PEG	Fe_3_O_4_ in water (suspension)	encapsulation of IONP into liposomes coated with PEG	90–110 (AFM)	-	[155]
MCIO/liposomes-PEG/PEG-Folic acid/Doxorubicin	Fe_3_O_4_ in water (commercial ferrofluid)	encapsulation of IONP and doxorubicin into the liposomes coated with PEG and PEG + folic acid	156 + −11 ^b^ 361 + −20 ^b^	-	[220]
MCIO/CaCO3 embedded	Fe_3_O_4_/(OA + OA) dispersed in water; aqueous ferrofluid	fast precipitation	5–6 µm ^a^	0.44–2.88	[167]
MCIO/Polymer-embedded colloidal assemblies	MnFe_2_O_4_/LA; Fe_3_O_4_/OA dispersed in toluene/tetrahydro furane	thermal decomposition/colloid destabilization by acetonitrile	70–134 ^a^	-	[65]
MCIO/CA obtained by fractionation	-	high temperature hydrolysis polyol approach	19.7–28.8 ^a^	65.4–81.8	[75]
MCIO	-	microwave irradiation	100 ^a^	38.3	[221]
	**Non-spherical particles/composites**				
Assembled supraparticles: mgPS, mgPVP and binary TiO_2_/mgPS	**Primary**: OA-stabilized Fe_3_O_4_ and CoFe_2_O_4_ ferrofluids **Secondary**: mgPS: SDS-stabilized mg/PS NPs and mgPVPs (aqueous magnetic colloids)	evaporation-induced assembly	supraparticle assemblies: mm range	52	[172]
Anisometric, ellipsoidal magnetic Janus supraparticles	Aqueous suspensions of Fe_3_O_4_@SiO_2_ core–shell NPs (20–30 nm)	evaporation-induced self-assembly	supraparticle assemblies: mm range	-	[171]
Magnetic halloysite nanotubes (HNT)	OA-coated Fe_3_O_4_ NPs (10 nm) in hexane (ferrofluid)	evaporation-induced self-assembly in the lumen of HNT (inner diameter 15 nm)	several hundreds of nm length magnetic HNTs	-	[175]
SMPs with dimpled and crumpled morphologies	Oleic acid-coated Fe_3_O_4_ NPs dispersed in hexane (ferrofluid)	emulsion-templated self-assembly	ìm range (average: 0.5 ìm)	-	[125]
2D and 3D mesocrystalline films	Oleic acid-coated Fe_3_O_4_ truncated nanocubes in toluene (ferrofluid) (≈10 nm–AUC, HRTEM, SEM)	self-assembly	-	-	[176]
Nanochains/nanobundles	ã-Fe_2_O_3_ NP clusters encapsulated within silica shells (114, 146 nm-TEM) (aqueous ferrofluid)	magnetic field-assisted self-assembly	nanochains of various lengths consisting of different no. of nanoclusters (6–40) Nanobundles: ≈1–2 μm wide and ≈5–10 μm long	15–45	[177]
Nanofibers	**Primary**: OA-capped Fe_3_O_4_ nanoparticles (8 nm-TEM) **Secondary**: SDS-stabilized PS-capped Fe_3_O_4_ NPs (127–237 nm-DLS) (aqueous dispersions)	magnetic field-assisted self-assembly	PS-Mag-H Nanofibers: 6.4 ± 2.5 μm (average no of NPs/fiber: 55) PS-Mag-J nanofibers: 3.0 ± 1.1 (average no of NPs/fiber:13)	84	[178]
Polymer-like chains and networks	SDS-stabilized Fe_3_O_4_-loaded PS nanospheres (80–350 nm-DLS) (aqueous dispersions)	magnetic field-assisted self-assembly	statistical, block copolymer, branched and network-like morphologies (micrometer length-scale)	-	[179]
Self-assembled elongated Janus NP clusters	Janus magnetic nanoparticles (≈20 nm) prepared by grafting (PSSNa) or (PDMAEMA) to the surfaces of negatively charged PAA-coated Fe_3_O_4_ NPs	pH-triggered self-assembly	elongated NP clusters with tunable NP number	-	[183]
Helical magnetoresponsive superstructures	Hydrophobic silica particles dispersed in octane based ferrofluid	emulsion evaporation-induced magnetic field assisted self-assembly	asymmetric dumbbell-type configurations (micrometer length scale)	-	[208]
Agar-encapsulated anisotropic microrod supraparticles	SPIO NPs (10 nm) (aqueous ferrofluid)	magnetic field-assisted self-assembly	iron oxide and silica-coated iron oxide microrod supraparticles (20 to 100 nm in diameter and 100 nm to 10 μm in length)	25–60	[184]
Anisotropic rodlike supraparticle structures	SPIO NPs (10 nm) (ferrofluid)	magnetic field-assisted self-assembly	anisotropic rodlike supraparticles diameters: 30 to 300 nm; length: 100 nm to 10 μm	-	[185]
3D self-assembled Fe_3_O_4_ NP layers on Si	Spherical, Fe_3_O_4_ NPs coated with OA (aqueous ferrofluid)	magnetic field-assisted self-assembly		-	[186]
Ellipsoidal superparamagnetic nanoclusters	Oleate-capped iron oxide nanoparticles (ferrofluid in octane)	emulsion electrospinning	ellipsoidal superparamagnetic Fe_3_O_4_ nanoclusters average cluster diameter: equatorial axis: 94 nm; polar axis: 250 nm - STEM)	~47	[180]
1D periodic magnetic NP arrays within electrospun polymer fibers	OA-coated iron oxide nanoparticles (18 nm) (ferrofluid)	magnetic field-assisted electrospinning	length of magnetic NP arrays: >1.5 μm	0.77	[204]
Helical nanocrystal superstructures	Fe_3_O_4_ cubic, rounded cubic, octahedral Fe_3_O_4_ nanocrystals, and Fe_3_O_4_-Ag heterodimeric particles (10–15 nm) (ferrofluids)	magnetic field-assisted self-assembly	large domains (up to 1 mm^2^) consisting of enantiopure helices	-	[209]
MHMS rods	Stearic acid-stabilized Fe_3_O_4_ nanocrystals (6 nm-TEM) dispersed in CHCl_3_ (ferrofluid)	self-assembly	MHMS length: ∼150–200 nm; diameter: from ∼50 to 60 nm.	2.49	[222]
1D arrays of SPIO NPs	Fe_3_O_4_ NPs, shell-functionalized with a dopamine sulfonate zwitterionic ligand and catechol-modified hydrophobic dye	Mg^2+^-mediated supramolecular polymerization	length: micrometer range	-	[223]
1D assemblies of Fe_3_O_4_ nanocrystals	Poly(4-styrenesulfonic acid-*co*-maleic acid) sodium salt-protected Fe_3_O_4_ nanocrystals (ferrofluid); ≈320 nm	ink-jet printing	1D NP assemblies length ≈31.0 µm	52	[214]
PAM hydrogel-encapsulated linear NP assemblies	15 nm single Fe_3_O_4_ NPs and 200 nm core–shell Fe_3_O_4_@carboxylated SiO_2_ nanospheres	magnetic field-directed assembly	linear NP assemblies with L/D ratio up to 10^2^–10^3^	~25	[94]
Stripe-like NP patterns	Primary γ-Fe_2_O_3_ NPs forming ≈230 nm clusters	magnetic field-directed assembly	micro-scaled size assemblies (20 μm wide and ~400 nm high)	-	[215]
Micro-sized NP assemblies of different geometries (cylinders, stars, triangles, cubes, etc.) forming chains on application of magnetic field	300 nm diameter silica-coated SPIO colloids	micro-lithography/magnetic field-assisted assembly	sub-5 micron superparamagnetic NP assemblies with variable shapes; micrometer-long magnetic field-mediated chain assemblies	-	[216]

**Size**: ^a^ TEM; ^b^ DLS. **Abbreviations**: CMD—carboxymethyldextran; CTAB—cetyl trimethylammonium bromide; PEI—poly(ethyleneimine); PAA—poly(acrylic acid); OA—oleic acid; OAm—oleylamine; SDS—sodium dodecyl sulfate; NIPAM—N-isopropylacrylamine; AA—acrylic acid; PEG—poly(ethylene glycol); PNIPAM—poly(N-isopropylacrylamine); PBMAg-C12—poly(isobutylene-alt-maleic anhydride) grafted with 1-dodecylamine; CA—citric acid; MCT—medium-chain triglycerides; PEG-DSPE—distearoyl-phosphoethanolamine-N(methoxy(polyethylene glycol)-2000); DTAB—dodecyltrimethylammonium bromide; PLGA—poly(lactic-co-glycolic-acid); GA-PEG-OH—gallol-bearing PEG; PS-*b*-PAA—poly(styreneblock-allyl alcohol); PScMA—poly(styrene-co-maleic anhydride); PS—polystyrene; SMP—superparamagnetic microparticles; PS—Mag-H magnetic polystyrene nanoparticles; PS—Mag-J-Magnetic Janus nanoparticles; AUC—analytical ultracentrifugation; PSSNa—polystyrene sodium sulfonate; PDMAEMA—polydimethylamino ethylmethacrylate; MHMS—Magnetic helical mesostructured silica; PAM—polyacrylamide; SPIO—superparamagnetic iron oxide.

The great variety of magnetic multi-core particles developed, starting usually from ferrofluids, illustrate the progress in the design and production of these versatile magnetic vectors with adjustable physicochemical properties (e.g., size, magnetic moment, surface charge, morphology, shell thickness), taking into account the requirements of achievable magnetic field strength and gradient, as well as of colloidal stability in biorelevant media [82,88,224,225]. At the same time, these results are indicative of the difficulty encountered so far in obtaining magnetic theranostic materials combining harmoniously all the critical properties for their effective application [43,81,147].

## 4. Structuring Processes Small-Angle Scattering Investigations

Small-angle scattering techniques using X-rays (SAXS) or neutrons (SANS)—commonly referred to as SAS techniques—are very useful for obtaining detailed structural information about particles and particle ensembles in the size range from 1 nm up to a few hundred nanometres. Such information is essential to link observed macroscopic properties, e.g., viscosity, elasticity, or optical properties, to the nanoscale structure. These methods also offer reliable information on colloidal stability and are sensitive to the onset and development of ordering in magnetic fluids [105,226,227,228,229]. One specific feature of SAS techniques is that the particle systems can normally be studied in their “natural” state—e.g., biologically relevant media—without the use of preparation methods that might otherwise disturb the structure and/or interaction between particles. On the other hand, it is also possible to play with the composition of the solvent (or matrix) surrounding the particles in order to enhance the signal from one or more particle components when studying non-homogeneous or composite particles. The latter is particularly interesting with SANS, where one can in many cases completely mask the contribution from selected components or enhance the contribution from others (isotope substitution). For the specific case of magnetic particles, SANS has the additional advantage that the neutron magnetic moment can be used to probe the magnetic particle structure. This is an important asset of the SANS technique that will be elaborated in the following. The X-ray variant (SAXS) has other advantages, such as very high flux and improved spatial resolution within the sample, making it a useful complementary probe, even for magnetic particles. Moreover, the combination of such advanced scattering methods with more standard techniques such as DLS, TEM, and DC magnetometry can provide very detailed characterization of magnetic nanoparticles and ensembles. Concerning DLS, it should be noted that sizes extracted by this method will generally include the effect of a solvation/hydration layer around the particles. The thickness of this layer can be relatively large, with the result that the sizes found with DLS sizes will generally be higher than those obtained from SAXS or SANS analysis.

The contrast obtained in a SAS experiment is governed by the distribution of scattering length density (SLD) in the system, which is determined by the density and structural organization of the atoms in the sample. Typically, a nanoparticle with a core of, for example, iron oxide will have a different SLD than the shell or coating surrounding it, and both will usually have SLD values different from the solvent. The SLD varies depending on the type of probe that is used. For X-rays, the SLD value reflects the density of electrons, and for neutrons, it reflects the average interaction distance (scattering length) over a certain volume. The strength of the scattering signal in a given experiment depends on these differences (to the second power) as well as on the shape/size of the scattering entities, which means that the size and shape of the various components in the system can be determined (at least in principle) via fitting to predefined mathematical models.

**Figure 15:**
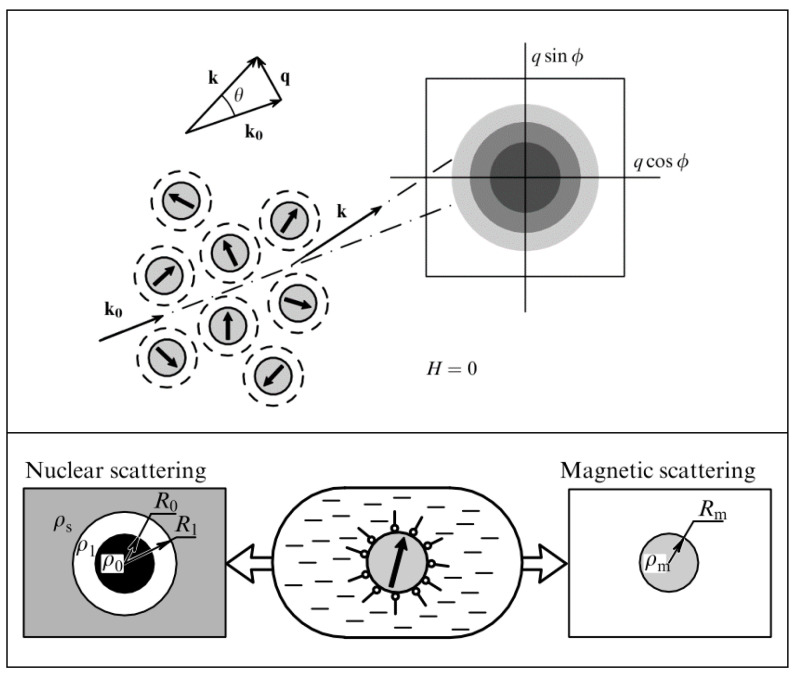
**Top**: Colloidal magnetic particles subjected to a beam with wave vector **k**_0_ in the absence of an external magnetic field. Magnetic moments of the particles have arbitrary directions, and the scattering pattern is isotropic. **Bottom**: Model representation for the nuclear and magnetic particle structures in a magnetic fluid. R_0_ is the physical size of the core, and R_1_ is the radius of the shell/coating. The parameters ρ_0_, ρ_1_, and ρ_s_ are the nuclear scattering length densities of the core, shell, and solvent, respectively. ρ_m_ is the scattering length density for the magnetic part, having a radius R_m_. (Reprinted with permission from [229]. Copyright Uspekhi Fizicheskikh Nauk 2010).

While X-rays interact with the electrons of the material, neutrons scatter from the nuclei of a material via the short-range strong nuclear force (nuclear scattering) but also from any unpaired electrons that exist in magnetic materials via dipole–dipole interactions (magnetic scattering), cf. Figure 15. The latter means that neutrons can be used to probe the magnetic structure in addition to other physical characteristics.

**Figure 16:**
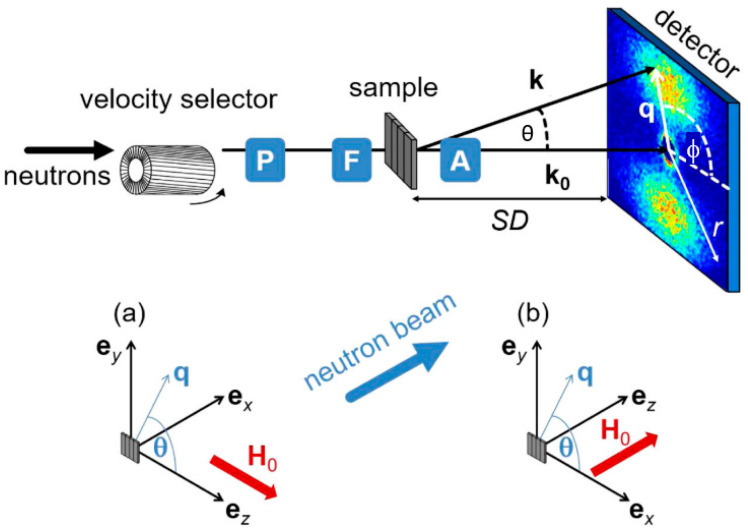
Schematic of a small-angle scattering technique using neutrons (SANS) setup and the two typical scattering geometries in magnetic SANS experiments, with **H**_0_ as the applied field. (**a**) Incoming beam (defined by **k**_0_) perpendicular to **H**_0_; (**b**) **k**_0_ parallel to **H**_0_. The symbols “P,” “F,” and “A” denote the polarizer, spin flipper, and analyzer, respectively. The angle φ describes the azimuthal anisotropy of the scattering pattern on a two-dimensional position-sensitive detector. In a standard (un-polarized) SANS experiment, P, F, and A are not present. In that case, the experimental setup resembles that of small-angle scattering techniques using X-rays (SAXS), apart from the fact that for SAXS setups, the wavelength is normally defined by a crystal monochromator. Adapted from [230]. Copyright 2019 by the American Physical Society.

When there is magnetization in a material, selection rules imply that the scattered neutrons are sensitive only to the component of the magnetization that is perpendicular to the so-called scattering vector **q** (cf. Figure 15, top). Here, **q** is a vector simply defined as **k**-**k_0_**, with **k**_0_ and **k** being the wave vectors of the incident and scattered neutrons, respectively. Both the incident and scattered neutrons (or X-rays in the case of SAXS) are assumed to have the same wavelength λ (elastic scattering). The scalar of **q** can be written as q = (4π/λ)sin(θ/2), where θ is defined as the scattering angle. With SANS, one can use polarized neutrons to isolate the magnetic scattering from the overall signal and determine the directional components of the magnetization. The magnetic scattering is in this way regarded as composed of two orthogonal components: perpendicular and parallel to an applied external field; see Figure 16.

In polarization-analyzed small angle neutron scattering (PASANS), the neutron polarization spin state is typically defined as either + or −. The neutrons coming toward the sample may be polarized by means of a supermirror and when needed, the initial neutron polarization can be reversed using a radiofrequency spin flipper. An incoming neutron that is polarized in one direction (+ or −) can make a spin-flip through interaction with the magnetic material and thus come out behind the sample with a − or + direction. This spin direction can be measured with a ^3^He-based neutron spin analyzer. Thus, there are four different scattering intensities (cross-sections) available, depending on the initial and final neutron spin, + +, + −, − +, − −, and measuring these makes it possible to extract the magnetic contribution from the sample. Scattering that takes place without a flip of the neutron spin, i.e., “+ to +” and “− to −” contains information about nuclear scattering plus the magnetic scattering from moments parallel to the applied field, whereas scattering with a flip of the neutron spin, “+ to −” and “− to +” contains only magnetic scattering. By inspecting the scattering data at specific angular positions on the 2D SANS detector (cf. Figure 16), the nuclear scattering can be subtracted from the total scattering, giving the net contribution from the magnetic part. However, the best way to treat the scattering data is normally a model-based fitting of the full anisotropic 2D-detector pattern containing the different angle-dependent contributions. Then, any magnetic contribution to the SANS signal found in this way will be a result of nanoscale variations either in the magnitude and/or orientation of the magnetization in the material.

It should be mentioned that information can be obtained also without a full polarization analysis, i.e., without the use of a spin analyzer (half-polarized cross-sections). Then, the intensity in the limit of the smallest scattering angles (q -> 0) is proportional to the magnetic moment of the particles and can be directly compared to macroscopic magnetization measurements. In some cases, the details can be difficult to extract with this method, especially if one has a system with a complex internal spin structure [230]. However, there is still the advantage, compared to non-magnetic SANS, that the contribution from incoherent or other background scattering is eliminated.

Magnetic small-angle neutron scattering can be used for a large variety of systems, i.e., permanent magnets, magnetic steels, skyrmion lattices, noncollinear spin structures and others [230]. However, in the present article, we look mainly on the applicability for colloidal magnetic nanoparticles. SANS can provide information both on the spatial distribution of magnetization within nanoparticles (intraparticle magnetization) as well as on superstructures or aggregates induced by dipolar interactions between particles (interparticle structure formation).

For non-interacting particles, the scattered intensity (SANS or SAXS) is basically proportional to the form factor *P* (*q*,*R*) for an individual particle. This is equivalent to saying that the structure factor for the system equals one. Then, interactions between different particles (interparticle interactions) in a sample can be observed as a deviation from 1 for the structure factor. Qualitatively, this is a straightforward way of separating attractive vs. repulsive interparticle interactions, and by data modeling, the type of interaction (e.g., magnetic dipole or electrostatic) can be clarified and the interaction parameters extracted.

For dispersions of magnetic nanoparticles or ferrofluids, the application of a magnetic field will typically result in an anisotropy of the Brownian motion in solution and a lowering of the concentration fluctuations along the field direction. This results in an anisotropic scattering pattern on the 2D detector, due to the anisotropic structure factor. In such cases, detailed analysis of the SANS data may give information of the structures formed. These can be short-range ordered aggregates but also chain-like structures that orient in the direction of the applied magnetic field [231,232,233], and even pseudocrystalline ordering has been observed [234]. For magnetic colloidal nanoparticles, in the modeling of the scattering patterns, one introduces a magnetic form factor *P*_m_ (*q*,*R*) in addition to the standard (nuclear) form factor *P* (*q*,*R*) that is employed in non-magnetic SANS studies. In addition, one has to account for the contrast (difference in scattering length density with respect to the surrounding material): Δρ_m_ for the magnetic part and Δρ_n_ for the nuclear part. These contrast factors are squared and multiplied with the form factor before integration over the particle to give the scattered intensity.

There are quite a few studies where SANS has been used primarily to study structure formation, aggregation behavior, and/or stabilization of magnetic particle systems [235,236,237,238,239]. For example, information has been gained on the use of different stabilization mechanisms for the magnetic particles, such as single steric, double steric/electrostatic, and ionic (electrostatic) surface coating [235,239]. In this case, valuable information, such as the size distribution and effect of stabilizers can be gained using standard SANS techniques, without the need for polarized neutrons. This is particularly true when contrast–variation experiments are done, since specific parts of the system (e.g., core or surface coating) can be highlighted. As an example, [238] studied magnetite nanoparticles stabilized by sodium oleate with and without the addition of polyethylene glycol (PEG). Then, different types of stable aggregates were described based on the SANS data, with the addition of PEG resulting in a reorganization of the structure of the aggregates, from initially small/compact aggregates of ca. 40 nm in size to large fractal-type structures above 120 nm.

For magnetic nanoparticles, the existence of a magnetically inactive or canted layer near the particle surface has been suggested in theoretical studies and via measurements of bulk magnetization [230]. Then, a lower saturation magnetization than for the bulk material is attributed to such surface spin disorder. This has led to a generally accepted model of magnetic nanoparticles as consisting of a superspin core and a surface region of canted or disordered spins. With polarized SANS, it is possible to obtain information on such a structure via extraction of the spatial distribution of magnetization within the nanoparticle. This can be done by looking at the difference between the nuclear and magnetic particle sizes together with the variation found in the magnetic scattering length density (ρ_m_) obtained through fitting of the observed 2D scattering data with an appropriate model.

**Figure 17:**
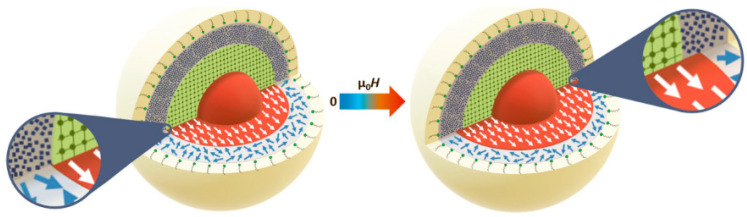
Schematic of the structural and field-dependent magnetic NP morphology: The vertical cuts represent the structural morphology, consisting of a structurally coherent grain size (green) and structural disorder (blue) within the inorganic particle (gray). The horizontal cuts represent the magnetic morphology, consisting of a collinear magnetic core (red) and spin disorder (blue) within the inorganic particle surface layer (gray). The particle is surrounded by an oleic acid ligand layer (beige). Structural and magnetic particle sizes are equal in zero field (left), whereas the initially disordered surface spins are gradually polarized in the applied magnetic field such that the magnetic radius increases beyond the structurally disordered surface region (right) (Reprinted from [240] under CC—Creative Commons Attribution 4.0 International license.).

According to the usual static picture, nanoparticles have a constant overall magnetic moment corresponding to the magnetic size. The magnetic core is surrounded by a surface layer where spin canting or spin disorder is present, and the thickness of this layer is considered to be independent of the particle size or applied magnetic field. For small particle sizes (below 10 nm for ferrofluids), the above situation would be responsible for a significant decrease of the overall magnetic moment of particles. In contrast to this picture, Zakutna et al. [240] by applying spin-resolved SANS demonstrate a significant increase of the magnetic moment of ferrite nanoparticles with an applied magnetic field in case of a toluene-based Co–ferrite ferrofluid (Figure 17). The data support a magnetic field-dependent noncorrelated surface spin disorder rather than spin canting at the particle surface. Thus, this information modifies the simplified picture of a fixed-size surface layer and illustrates the high capabilities of small-angle scattering techniques to elucidate structural details at the nanoscale.

As another example, Hoell et al. [241] utilized half-polarized SANS to investigate ferrofluids based on Ba–ferrite particles with oleic acid as the surfactant and dodecane as the carrier liquid. With half-polarized SANS, the measured intensity is *I*^+^(Q) or *I*^−^(Q), depending on the polarization state of the incoming neutron. For a particle built up by a magnetic core surrounded by a non-magnetic organic surfactant layer, the scattering contrast for the magnetic core is Δρ_core_= (ρ_n_ ± ρ_m_) − ρ_solvent_; i.e., it is dependent on the polarization. On the other hand, that of the shell is Δρ_shell_ = ρ_n_ − ρ_solvent_, independent of the polarization, allowing for separation of the contributions from the core and the shell. In addition, isotopic H/D contrast variation of the carrier liquid was used to better separate the entities present in the solution. Thus, the existence of a core–shell structure could be clearly verified, where the shell of surfactants was found to be near impenetrable for the carrier liquid. Furthermore, the data revealed magnetic aggregates as well as isolated surfactant molecules.

Kons et al. [242] also used half-polarized SANS but combined with X-ray magnetic circular dichroism (XMCD) spectroscopy to investigate the distribution of magnetization in heterogenous magnetic nanoparticles consisting of a metallic iron core and iron oxide shell. The particles were studied as a powder, not in a suspension. Modeling of the polarized neutron scattering showed large variations in the magnetization distribution radially, with a region of reversed magnetization adjacent to the metallic core. It was suggested that the interfacial roughness plays a role in the development of this magnetization profile.

Recently, Brok and coworkers [243] showed that the technique can be developed even further by introducing so-called phase-sensitive small-angle neutron scattering (PS-SANS) to gain information specifically about the particle coating. They studied particles consisting of Fe_3_O_4_ cores (25 nm diameter) coated with a layer of oleic acid, a layer of amphiphilic polymer, and finally a layer of polyethylene glycol. Here, the magnetic core with a known radius *R*_m_ and scattering length density ρ_m_ served as the reference, whereby measurements with polarized neutrons, in combination with finite element analysis, could be used to determine the SLD distribution and thus the detailed structure of the polymer coating.

Oberdick et al. [244] studied Fe_3_O_4_/Mn_x_Fe_3−x_O_4_ core/shell nanoparticles of ca. 7 nm diameter size with a 0.5 nm Mn–ferrite shell. The polarized small angle neutron scattering of dried powders of these particles demonstrated both parallel and perpendicular magnetic correlations, suggesting multiparticle coherent spin canting in an applied field. Their results illustrate how magnetic core/shell nanoparticle systems have a potential to be engineered for spin canting across the whole of the particle instead of only at the surface.

Fu and coworkers [245] used polarized SANS to study the assembly of core–shell iron oxide magnetic nanoparticles (dispersed in toluene) induced by magnetic field. These authors also followed the formation of large-scale nanoparticle aggregates, using neutron scattering at very low scattering angles (VSANS). Specifically, a three-dimensional long-range ordered superlattice of iron oxide NPs with a face-centred cubic (fcc) crystal structure was found to exist already at moderate fields (above 0.02 T). For investigating the formation of very large structures, above 100 nm or so, the use of VSANS-type setups are highly beneficial. These are now becoming available at several large-scale facilities around the world.

**Figure 18:**
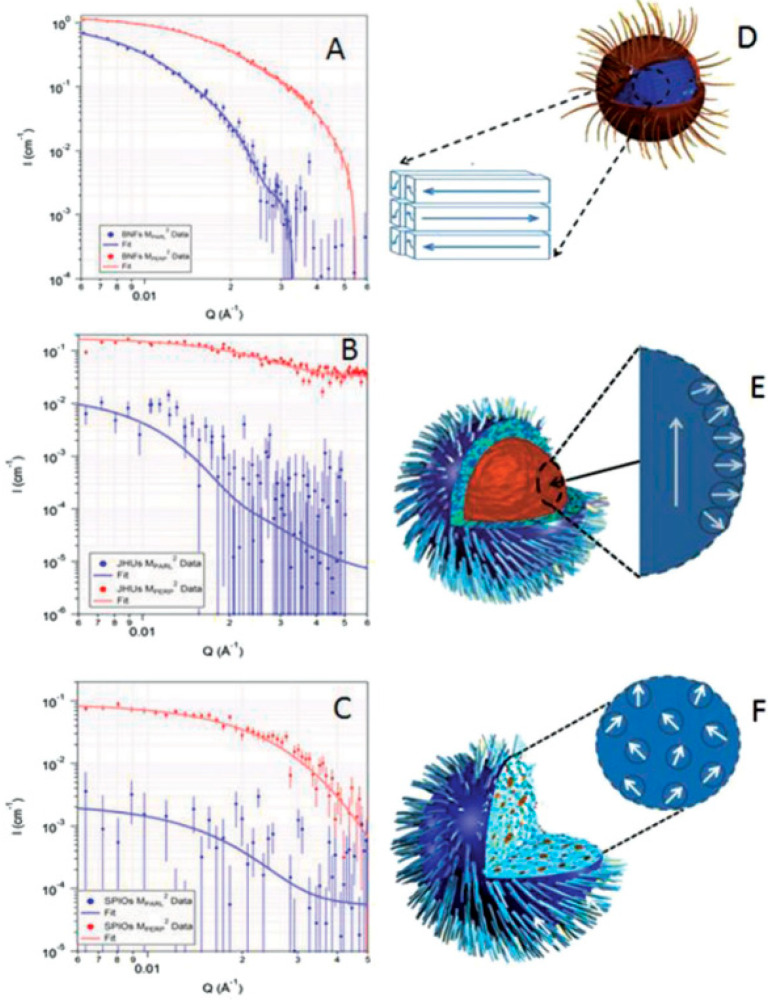
Magnetic scattering contributions parallel (blue) and perpendicular (red) to the guide field obtained with polarized SANS measurements on three different dextran-coated magnetic particles in D_2_O at room temperature: (**A**) BNF particles (Bionized nanoferrite); (**B**) JHU particles (Johns Hopkins University); (**C**) nanomag-D-spio (SPIO) nanoparticles. Continuous lines are model fits to the data. To the right is shown domain structures of: (**D**) BNF; (**E**) JHU; (**F**) SPIO particles obtained from analysis and modeling of the polarized SANS data. Republished with permission of John Wiley and Sons, from [246]; permission conveyed through Copyright Clearance Center, Inc.).

Dennis et al. [246] used polarized SANS on three different colloidal magnetic particle systems suspended in water, all having a core with a mixture of Fe_3_O_4_ and γFe_2_O_3_ and a shell of dextran, with a mean overall (hydrodynamic) diameter around 100 nm, cf. Figure 18. Through analysis of the polarized SANS data, the internal magnetic structure could be characterized in detail. For the first particle type (BNF), synthesized through high-temperature, high-pressure homogenization, a dense core consisting of stacked parallelepiped-shaped crystallites (8 × 26 × 66 nm) was found, cf. Figure 18D. Dipolar coupling between the crystallites favor the alignment of the collinear components of the magnetic moments along the same direction, while the side-by-side components of the moments arrange antiparallel to one another. For the second particle type (JHU), synthesized with high-gravity controlled precipitation, the core was found to consist of several near spherical crystallites of 16 nm diameter. Magnetically, this core can be considered to have a core–shell-like structure with a magnetic core of 36 nm in diameter magnetized parallel to the guide field and a shell with average thickness of about 7 nm that is broken into smaller subdomains magnetized perpendicular to the guide field (Figure 18E).

For the third particle type (SPIO), nuclear scattering showed that the core consisted of a collection of approximately spherical crystallites of ca. 9 nm diameter, which were dispersed throughout the polymer matrix of overall diameter ca. 100 nm, Figure 18F. Analysis of the magnetic scattering shows spherical domains of 14 nm in diameter, which is consistent with an average cluster size of 2–3 crystallites, as also suggested from transmission electron microscopy data.

Recently, Bersweiler et al. [247] took the analysis a step further by including Bayesian analysis in the characterization of magnetic nanoparticles. These were spherical iron oxide nanoparticles synthesized by a high-temperature thermal decomposition coated with a silica shell. First, a standard least-squares fit procedure was used to obtain an initial fit to the polarized SANS and magnetometry data, followed by a Bayesian approach to accurately refine the parameters. The advantage with a Bayesian analysis is that one gets a direct visual feedback on the quality of the fit, which prevents overfitting and incorrect results and is especially useful in the case of highly correlated parameters.

As commented earlier, SAXS does not give direct information about magnetic structure, and it has therefore been mainly employed to study the aggregation behavior of magnetic nanoparticles. For example, Coral-Coral and Mera-Córdoba [248] used SAXS on different aqueous colloidal suspensions of citric acid-stabilized magnetite nanoparticles to extract the particle size distribution and aggregation states. Ramified chain-like aggregates were described for the better-stabilized sample, whereas a more compact structure was found for the less-stabilized sample. The size distribution obtained via SAXS models were found to be in good agreement with that determined using TEM. However, this may differ depending on the system, since TEM probes a small selected part of the material, while SAXS (and SANS) probes the average structure of the whole particle population.

Paula [249] also used SAXS to investigate the local colloidal structure of a ferrofluid in the presence of an external magnetic field. The nanoparticles were of the core–shell type, with a core of manganese ferrite and a maghemite shell. Two levels of structure could be described, both clusters and isolated particles, with and without applied magnetic field. A combination of analysis methods was used to extract detailed results: fitting to experimental data with the so-called Beaucage unified model, analysis of the radial distribution function, as well as theoretical calculation of the radius of gyration as a function of the moment of inertia.

**Figure 19:**
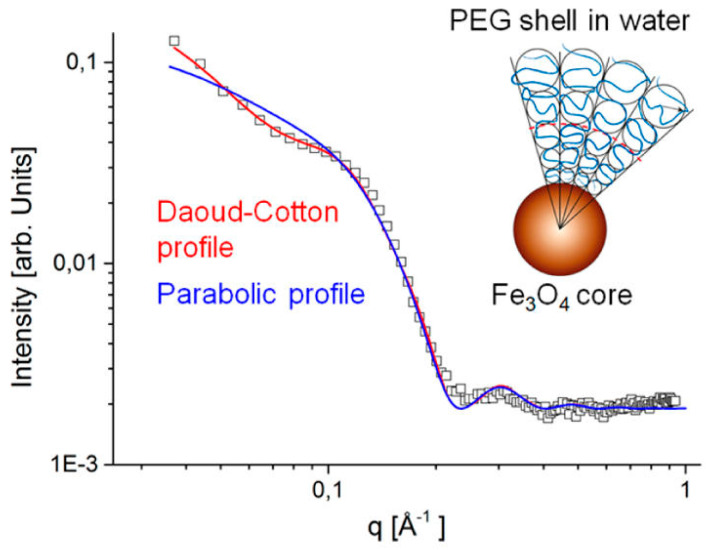
Shell density profile for PEG-coated iron oxide nanoparticles determined by SAXS. (Reprinted with permission from [250]. Copyright 2015 American Chemical Society).

SAXS can also be useful to investigate the local structure, specifically the structure of the polymer shell that is important for particle stabilization. Grunewald et al. [250] employed SAXS to investigate poly(ethylene glycol)-coated iron oxide NPs with high polymer grafting density and found that the density profile of the shell coating was well described by the Daoud−Cotton model [251], as shown in Figure 19. The result indicates a high constant density region of PEG close to the magnetic core and a decrease of PEG density according to r ^−4/3^ in the outer part of the shell. The data yields a high grafting density of ≈3.5 chains/nm^2^, explaining the excellent colloidal behavior of the investigated system.

**Figure 20:**
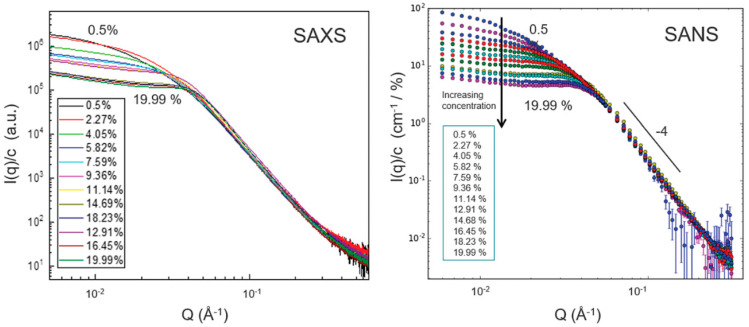
SAXS and SANS scattering patterns for citric acid-stabilized Fe_3_O_4_ particles at varying concentrations, normalized to the particle concentration. The SANS data have been background-subtracted for the H_2_O contribution. (Reproduced from [233] with permission from The Royal Society of Chemistry).

However, due to the complementarity of the X-ray and neutron techniques, the combined use of SAXS and SANS on the same system can be very useful to gain a good understanding of the structure and behavior of interacting magnetic particles [233,252,253]. For example, Vasilescu et al. [233] employed both SAXS and SANS to investigate water-based colloids of iron oxide magnetic particles with two different stabilization mechanisms—electrostatic (with citric acid) and electrosteric (with oleic acid double layer)—over a large concentration range. Important differences on the microscopic level that affect the interaction and stability of the magnetic fluids could be described in detail by the combined use of SAXS and SANS, as reflected in the scattering patterns (Figure 20). For this system, the electrostatic stabilization ensured good colloidal stability up to 30% hydrodynamic volume fraction and very high magnetization (78 kA/m), whereas the electrosteric stabilization showed the formation of relatively large clusters at lower volume fraction values.

Bender et al. [253] used a combination of SAXS and SANS together with static light scattering (SLS) on a colloidal dispersion of iron oxide nanoparticle cores (9 nm) embedded in polystyrene spheres (160 nm total diameter). Here, an indirect Fourier-transform method was used to extract the pair distance distribution function based on scattering data from all three techniques. The result showed that the cores were not homogeneously distributed but accumulated toward the surface layers of the polystyrene spheres. These authors also applied an indirect Fourier-transform to magnetization data, finding two distinct peaks in the moment distribution. The main peak corresponded to the intrinsic moment distribution of individual non-interacting iron oxide nanoparticle cores, whereas the second peak could be attributed to weak dipolar interactions. Furthermore, an increased susceptibility, i.e., shift to higher moment values, was found for particles dispersed in water compared to dry particles. This was interpreted as the formation of finite remnant moments due to the coupling of the spins of the cores inside some of the multi-core particles. In colloidal dispersion, the particles can rotate in the field direction, which could explain the increased susceptibility.

There have been relatively few SAS studies so far on multi-core magnetic particles. Eberbeck et al. [66] made use of SANS to investigate dextran coated multi-core magnetic iron oxide nanoparticles. In this case, the magnetic component of the scattering was found by subtracting the data along the detector axis parallel to the applied field (composed only of nuclear scattering) from the data perpendicular to the field (containing both the nuclear and the magnetic components). The nanoparticles were dispersed in D_2_O for measurements with field (0.25 T) to highlight the magnetic scattering, but also in H_2_O for measurement without field to highlight the nuclear scattering. The hydrodynamic diameter based on DLS was 106 nm, whereas TEM indicated iron oxide crystals with between 3 and 8 nm. This multi-core structure could be confirmed by SANS, showing parallelepiped shaped particles with 6.8 nm short axis. Furthermore, the SANS measurements showed that the magnetic size was smaller than the physical size. In this way, one could identify a surface layer, slightly above 1 nm thick, where the atomic magnetic moments do not align with the magnetization within the core.

Szczerba et al. [254] used SAXS to study the structure of both single-core and multi-core iron oxide nanoparticles with oleic acid used as a surface coating. Although the magnetic structure is not possible to determine with SAXS, the method is very useful to get accurate information about the shape and size distribution of the cores and about how cores are clustered in multi-core particles. It was found that in the multi-core particles, the cores were arranged as a quite dense mass fractal cluster/network (fractal dimension of 2.9), but with the cores well separated from each other by the organic shell. The radii of gyration of the mass fractals could also be found, and the amount of primary particles in each cluster could be determined (117 or 186 depending on the preparation method).

**Figure 21:**
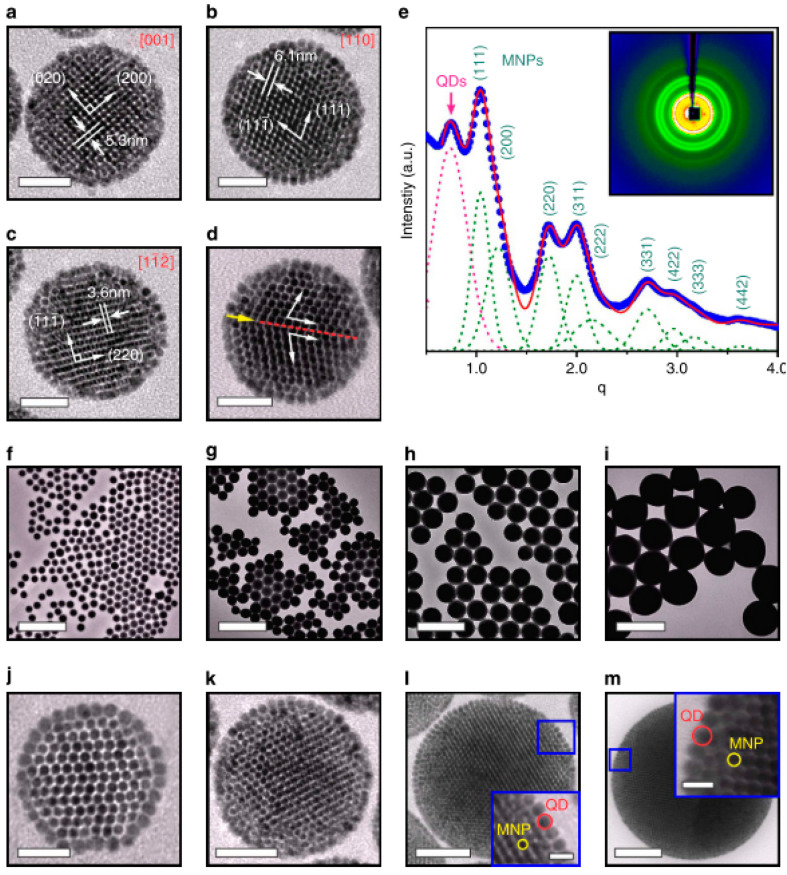
Supercrystalline CS-SPs (core–shell supernanoparticles) and their size-controlled syntheses. TEM images of supercrystalline CS-SPs viewed along different zone axes (**a**) (0 0 1), (**b**) (1 1 0) and (**c**) (1 -1 -2) (**d**) TEM image of a CS-SP with a stacking fault marked with a yellow arrow. Scale bars, 50 nm (**a**–**d**). (**e**) The integrated data from the SAXS pattern (inset) of CS-SPs show a position ratio series of q/q0 = 1/√(4/3)/√(8/3)/√(11/3)/√(4)/√(19/3)/√(8)/√(9)/√(12) (q0 is the position of the (111) peak) indicating an fcc close packing of the MNPs. Large-area TEM images (**f–i**) and higher magnification TEM images (j–m) of CS-SPs with an average diameter of 80 ± 9 nm (**f**,**j**), 120 ± 13 nm (**g**,**k**), 235 ± 30 nm (**h**,**l**), and 360 ± 60 nm (**i**,**m**). The insets in l and m are the position of the (111) peak, q = 4πsinθ/λ, indicating an fcc close packing of the MNPs. Large-area TEM images (**f**–**i**) and higher-magnification TEM images zoomed-in images of the blue squares. Scale bars, 500 nm (**f**–**i**). Scale bars, 30 nm, 50 nm, 70 nm and 100 nm (**j**–**m**), respectively. Scale bars, 15 nm (insets of l and m). Red and yellow circles indicate the positions of QDs and MNPs, respectively. (Reprinted by permission from Copyright Clearance Center: Spinger Nature, Nature Communications, [144], Copyright 2014).

Chen et al. [144] employed SAXS to study so-called magneto-fluorescent core−shell supernanoparticles, cf. Figure 21. These have a core made of close-packed magnetic nanoparticles surrounded by a shell of fluorescent quantum dots and are coated with a thin layer of silica for structural support. With high-resolution synchrotron SAXS, it was possible to accurately determine the structure of the close-packed particles in the core. The first broad SAXS peak (cf. Figure 21e) represents diffuse scattering from the randomly distributed shell of quantum dots (QDs), while the other SAXS peaks can be assigned to Bragg peaks from an ideal fcc superlattice corresponding to the Fm3m space group, with a lattice constant of 10.4 ± 0.2 nm, which is consistent with TEM observations (Figure 21a–d).

Interparticle spacing was found from SAXS to be 7.3 nm, and given the MNP size of 5.9 nm, an average interparticle distance of 1.4 nm could be determined. This result is consistent with a situation where the oleate ligands on the MNPs surfaces are coiled and intercalated. In this work, Chen and coworkers [144] also showed that after surface PEGylation, such nanoparticles can be magnetically manipulated inside living cells while being optically tracked.

Very recently, Bender et al. [255] looked at so-called “magnetic nanoflowers”, which are defined as densely packed aggregates of superferromagnetically coupled iron oxide nanocrystallites. Polarized SANS was used to investigate the moment coupling within a powder of such nanoflowers. In a powder sample, these nanoparticles will agglomerate to clusters, and it was shown that the moments of neighboring nanoflowers tend to align parallel to each other. The overall system resembles a hierarchical magnetic nanostructure with three distinct levels, (i) ferrimagnetic nanocrystallites as building blocks, (ii) superferromagnetic nanoflowers, and (iii) supraferromagnetic clusters of nanoflowers. The authors suggest that the supraferromagnetic coupling within this system explains the enhanced magnetic hyperthermia performance observed for interacting nanoflowers.

It is important to note that one can also combine SANS (or SAXS) on dispersions of magnetic particles with rheological measurements to follow the so-called magnetoviscous effect (MVE). This very powerful combination of tools (Rheo-SANS/SAXS) is ideal to explore in situ the coupling between nanostructural features and macroscopical behavior (viscosity or viscoleasticity). In specialized setups, it is possible to vary independently both the magnetic field strength (as well as orientation) and the shear rate, providing large sets of data to follow the internal reorganization taking place for the magnetic particles [256,257].

**Figure 22:**
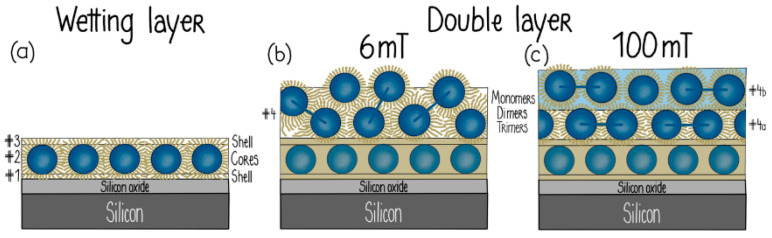
Schematic drawing of magnetic nanoparticle (NP) ordering determined for the first layers above the silicon surface, based on results from SANS and PNR. (**a**) Wetting layer, (**b**) double layer on top of the wetting layer in a magnetic field of 6 mT, and (**c**) double layer on top of the wetting layer in a magnetic field of 100 mT. The magnetic field is directed parallel to the surface. (Reprinted with permission from [186]. Copyright 2018 American Chemical Society).

Finally, it should be mentioned that in recent years, the technique of neutron reflectometry (NR) has shown to be very useful to probe magnetic particle configurations on solid surfaces. One can make use of polarized beams also in neutron reflectometry (PNR), and in this way extract both density and magnetization depth profiles near the surface [187]. This is particularly interesting for studying effects of particle coating and applied magnetic field on the self-assembly process of magnetic particles on surfaces with different hydrophobic/hydrophilic character. As a recent example, Theis-Bröhl et al. [186] used neutron reflectometry to perform a detailed characterization of the organization of monodisperse colloidal magnetite nanoparticles (NPs) onto silicon surfaces. Prior information about the internal NP structure and their interactions (e.g., dimer and trimer formation) was obtained from SANS measurements and used as input to the modeling of the neutron reflectometry data. The reflectometry results showed how the NPs assemble into close-packed layers on the surface followed by more loosely packed layers above (Figure 22). For layers in which the NPs are relatively free to rotate, the easy axis of the NP can readily orient along the field direction. In more dense packing, free rotation of the NPs is hampered, and the NP ensembles are thought to build up quasi-domain states to minimize energy, leading to lower magnetization in those layers.

**Figure 23:**
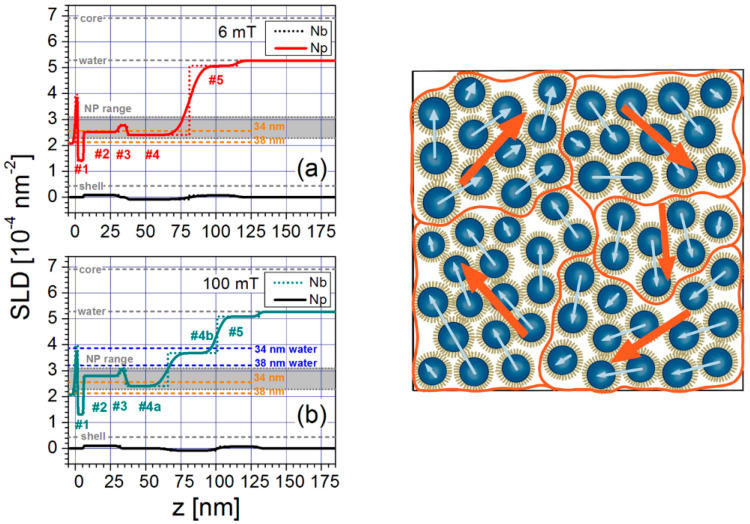
**Left:** Profiles of nuclear (Nb) and magnetic (Np) scattering length density (SLD) plotted as a function of distance z from the silicon surface determined from fits to polarized neutron reflectometry (PNR) data taken at 6 mT (**a**) and 100 mT (**b**). For comparison, SLD values for the magnetite core, water, and shell material are included as gray dashed lines. The SLD range between the compressed and stretched ligand model for isolated NPs is shown as a gray area. Model SLD values for a close-packed layer of truncated particles with shell material in the intershell gaps (orange dashed lines) and with water in the intershell gaps (blue dashed lines) are given for core/shell NP diameters of 34 and 38 nm, respectively. **Right:** Sketch of a possible magnetic moment distribution within an NP layer when the NPs experience a quasi-domain configuration. The solid orange outlines represent the domain walls. (Reprinted with permission from [186]. Copyright 2018 American Chemical Society.).

The primary information coming from reflectometry is the scattering length density (SLD), and the SLD profile as a function of distance from the surface is shown in Figure 23 (left) for this system. Here, one can clearly see the variation in SLD value with position, corresponding to the different layers indicated in Figure 22. The accurate absolute value extracted for the SLD (cf. y-axis) is extremely useful to identify different types of ordering with respect to the surface. As a result of dipolar coupling within each layer, the authors suggest that NPs may order in a quasi-domain structure, as shown schematically in Figure 23 (right).

The use of NR is relevant also to explore the stability of colloidal magnetic particles when exposed to surfaces, as compared to the bulk situation. Such differences should be taken into account in the stability requirements of these systems for long-term storage due to the interaction of the particles with container walls under different conditions. Avdeev et al. [258] employed NR to look for the adsorption of magnetic nanoparticles from highly stable (non-oversaturated) magnetic fluids onto silicon surfaces. The system studied was oleic acid-coated magnetite particles dispersed in a non-polar organic solvent (deuterated benzene) as well as a polar solvent (heavy water). The reflectivity data showed the formation of just one well-defined adsorption layer of nanoparticles at the interface in both cases. This layer was also insensitive to the effect of the external magnetic field but with the particle concentration in the benzene-based fluid being higher in the vicinity of the silicon surface as compared to the bulk distribution. For the water-based system, despite the presence of an aggregate fraction in bulk, the adsorption layer consisted of only non-aggregated particles.

Overall, detailed analysis of polarized neutron reflectometry data together with small-angle scattering measurements and model calculations of the arrangement of the NPs within the layers can provide a full characterization of the core/shell NP dimensions, degree of clustering, arrangement of the NPs within the different layers, as well as the magnetization depth profile.

In conclusion, SAS methods and reflectometry techniques, especially in combination with various in situ techniques, are found to be very useful for the study of soft matter in general and magnetic nanoparticles in particular. The continuously increasing interest for such setups at large-scale facilities, e.g., the two SANS instruments currently under construction at the upcoming European Spallation Source (ESS), is a clear demonstration of this.

## 5. Magnetic Behavior

The properties of multi-core magnetic composites (MMCs) need to be tailored to fulfill the requirements of the envisaged application. E.g., for drug targeting and magnetic separation applications, MMCs need to have no spontaneous magnetic moment, i.e., zero magnetic moment in the absence of an external magnetic field, in order to prevent spontaneous clustering, and an as high as possible magnetic field-induced magnetic moment in order maximize the magnetophoretic force.

The magnetic properties of MMCs are strongly influenced by both the magnetic properties of the constituent nanoparticles and their packing degree [259,260,261,262]. Magnetic nanoparticles are most often magnetic monodomains, therefore showing a permanent magnetic moment. Depending on the blocking temperature, the magnetic moment at the application temperature—room or body temperature—may be free or frozen inside the MNP. In soft magnetic nanoparticles, with zero or very weak magnetic anisotropy (crystalline, shape or surface anisotropy), i.e., with very low blocking temperature, the magnetic moment at room temperature is free to rotate inside the nanoparticle, and therefore, it is in permanent thermal fluctuation. Hard magnetic nanoparticles on the other side, with very high blocking temperature, have their magnetic moment frozen inside the nanoparticle at room temperature with its direction parallel to the strongest magnetic anisotropy axis, and therefore, very high temperature or strong magnetic fields are needed to rotate the hard MNP magnetic moment with respect to the nanoparticle. Consequently, when a magnetic field is applied, the particle magnetic moment will rotate via either the Brownian or Néel process or some combination of both, although the faster mechanism will typically dominate [263,264]. The rotational dynamics of magnetic nanoparticles in magnetic fields and the corresponding time-scale, as well as the collective magnetic behavior of magnetic nanoparticle systems, are important for most of the biomedical applications [260,262,265]. Due to the high packing fraction of MNPs inside the MMC, the magnetic dipole–dipole interaction may lead to a spontaneous MMC magnetic moment. The dependence on the external field **H** of the magnetic moment vector **μ(H)** is important for the derivation of the magnetophoretic force **F** = (**μ(H)**⋅∇)**H** [266].

**Figure 24:**
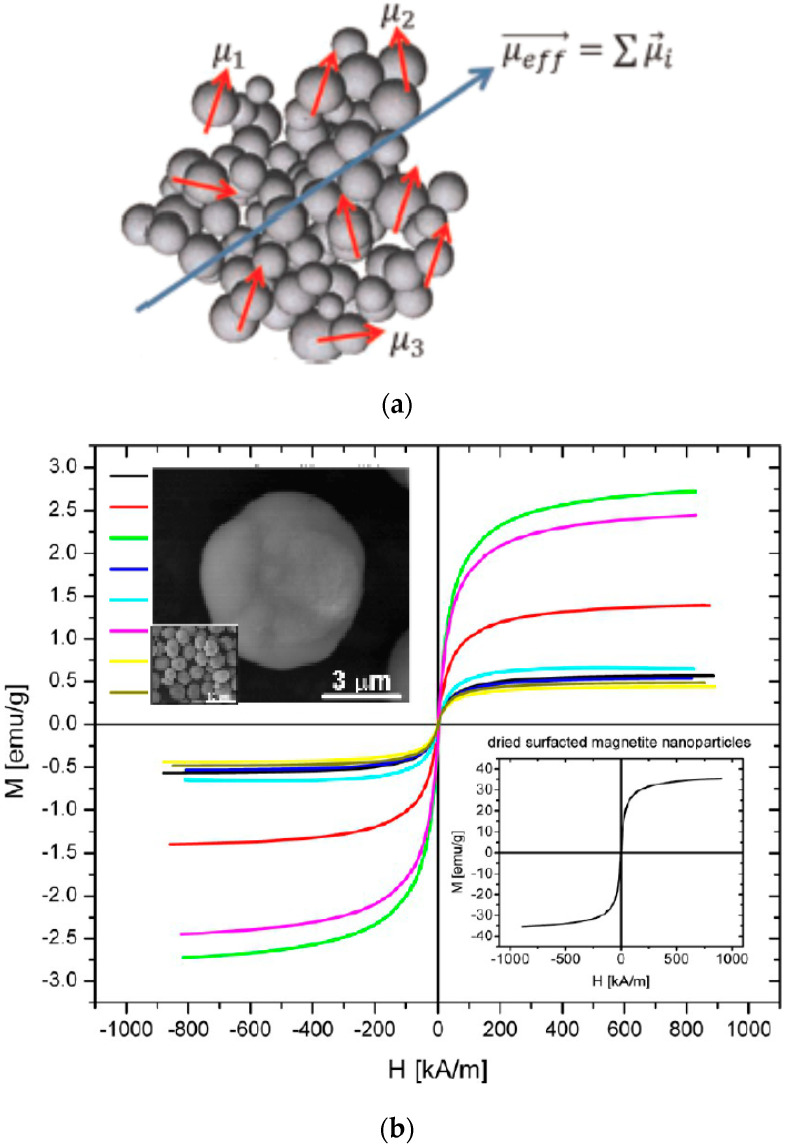
(**a**) Magnetic multi-core particle (reprinted from [77], Copyright 2015, with permission from Elsevier), and (**b**) Magnetization curves of the dried composite microparticles. Inset shows the magnetization of the dried surface coated magnetite nanoparticles. (Reprinted with permission from [167]. Copyright 2013 American Chemical Society.).

MMCs are clusters of closely packed and mechanically frustrated magnetic nanoparticles. Therefore, from the magnetic point of view, the MMC is a highly dense system of magnetic moments (Figure 24a).

In the case of small magnetic moment soft nanoparticles, the MMC’s magnetization can be understood with the help of the Langevin model [266] or, taking into consideration weak magnetic interparticle interactions [259], Ivanov [267] and Szalai [268] models. The magnetic moment μ of the MMC has a Langevin-like dependence on the applied magnetic field H: μ = μ(H) (Figure 24b). The magnetization **m**(**H**), i.e., volume-specific magnetic moment (**m**(**H**) = **μ**(**H**)/μ_0_/v [266]), is zero in the absence of the external magnetic field (m(0) = 0) and asymptotically reaches the saturation magnetization in strong magnetic fields. Due to MNP rotation-free magnetic moments and weak interparticle interactions, the MMCs have no coercive field or remnant magnetization.

In MMCs made of large soft magnetic nanoparticles, the synergy between the strong magnetic dipole–dipole interactions and magnetic moment rotational freedom may allow for a spontaneous non-zero resultant magnetic moment. As a result, dry dispersions of such MMCs have a non-zero coercive field and remnant magnetization. Such MMCs in liquid dispersions will have the tendency to cluster due to magnetic attraction. Under the action of an external magnetic field, the resultant magnetic moment will further increase due to magnetic–dipole interactions. As an example, in the polarized SANS investigations by Bender and coworkers on ensembles of ≈50 nm magnetic nanoflowers made of 5–15 nm soft magnetic nanoparticles, referred to previously, they discovered a hierarchical magnetic nanostructure consisting of three distinct levels [255].

MNCs made of hard MNPs will exhibit spontaneous magnetic moments either due to magnetic dipole–dipole interactions during solidification if the nanoparticles are large enough [77], or in case that the clustering is done, in an external magnetic field. In both situations, increasing or rotating the magnetic moment relative to the cluster will require a very high external magnetic field intensity.

The determination of an MMC magnetic moment μ(H) is not a trivial task. Experimentally, one usually measures the magnetic moment, whence the mass or volume magnetization may be obtained, of a size polydisperse MMC powder. Figure 24b shows the magnetic field dependence of the mass magnetization measured on a dry sample of calcium carbonate/magnetite/chondroitin–sulfate MMC [167]. Other than observing the features of a soft MNP ensemble, i.e., a lack of coercivity and Langevin-like field dependence, the determination of an MMC magnetic moment would require precise knowledge of the MMC mass statistics, which is difficult to obtain.

**Figure 25:**
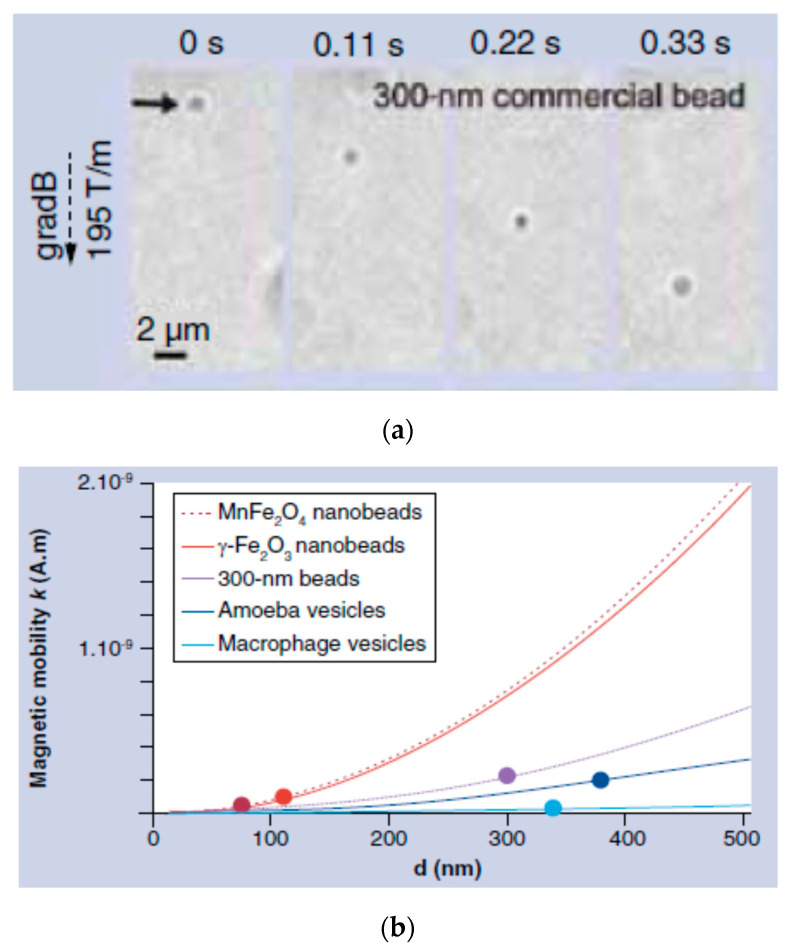
(**a**) Multi-core magnetic composite (MMC) moving in the 195 T/m zone, and (**b**) magnetic mobility of several types of MMC. (Reprinted by permission from Copyright Clearance Center: FUTURE MEDICINE LTD, Nanomedicine, [80], Copyright 2012).

Optical microscopy investigations can be used to determine the MMC’s magnetic moment [80]. Silva and coworkers [80] used a nickel nanorod and a strong permanent magnet to create a 195 T/m magnetic field gradient inside a flat optical cell. The B-field was computed numerically. From the time-sampled optical microscopy images (Figure 25a), the velocity v and hydrodynamic diameter d_h_ of the MMC is obtained. Using the velocity and the MMC hydrodynamic diameter, the magnetic mobility (k = μ/(3πd_h_)) is calculated (Figure 25b), whence the magnetic moment μ follows straightforward. The sole inconvenience of this method is the very narrow field values at which the MMC magnetic moment can be determined.

DC magnetization data were used by Bender and coworkers to determine an MMC magnetic moment (Bender et al. 2018c). Single and bimodal distributions of magnetic moments in the range of 10^−20^–10^−16^ Am^2^ were determined in FeraSpin-R fractionated MMC colloids (to be discussed later in this section). AC susceptibility measurements can also be used for the determination of MMCs’ magnetic moments [77,79]. Ahrentorp and coworkers [77] used TEM, AC susceptibility, and DC magnetization measurement data and suitable theoretical models to determine the effective magnetic moment of BNF Starch and FeraSpin R MMCs: 11.9 × 10^−18^ Am^2^ and 6.5 × 10^−18^ Am^2^, respectively. The data analysis revealed that in FeraSpin R MMCs, the interparticle magnetic interactions are stronger than in BNF Starch MMCs. The inconvenience of both methods is that only the spontaneous magnetic moment can be determined.

Determination of the MMC magnetic moment can also be done theoretically based on MNP size, morphology statistics, and packing information obtained from TEM.

**Figure 26:**
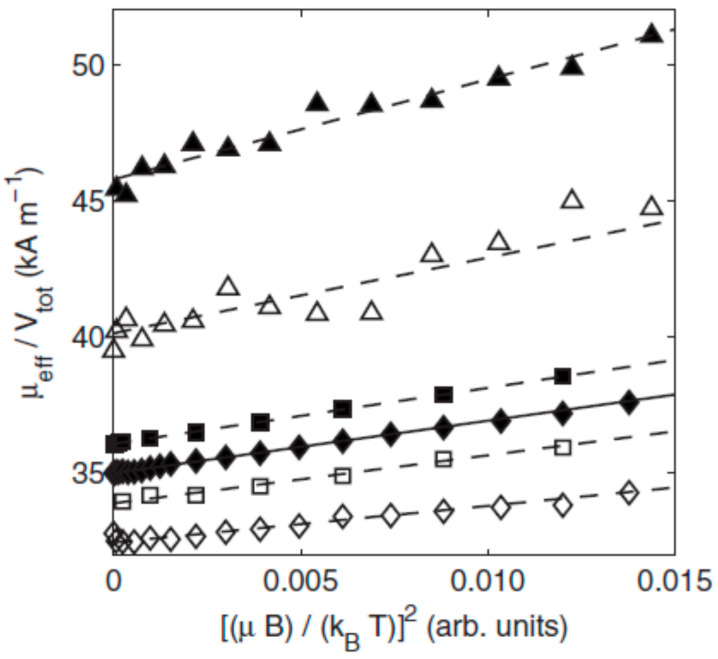
MMC effective magnetic moment dependence on the field induction square for several types of constituent nanoparticles: particle rotation in the liquid and interaction with the external field (filled diamonds), particle rotation in the liquid, interaction with the external field and lognormal size distribution of the MMCs (filled squares: D_m_ = 12 nm and σ = 1 nm, filled triangles: D_m_ = 12 nm and σ = 3 nm), particle rotation in the liquid, interaction with the external field and dipole-dipole interactions between the MMCs (open diamonds), and particle rotation in the liquid, interaction with the external field, log-normal size distribution of the MMCs and , interaction with the external field and dipole-dipole interactions between the MMCs (open squares: D_m_ = 12 nm and σ = 1 nm, open triangles: D_m_ = 12 nm and σ = 3 nm). (Reprinted figure from [269]. Copyright 2009 by the American Physical Society.)

Schaller and coworkers [269] performed analytical and numerical Monte Carlo simulations in order to determine the effective magnetic moment of MMCs composed of magnetic uniaxial and size mono- and polydisperse nanoparticles in weak magnetic fields. A polynomial quadratic field dependence of the effective MMC magnetic moment was found (Figure 26), whose coefficients (the free term standing for the spontaneous magnetic moment) were found to depend on MNP magnetic anisotropy, size statistic, and domain magnetization. The magnetic dipole–dipole interactions among the constituent nanoparticles diminishes the effective magnetic moment while increasing the diameter increases the effective magnetic moment. The effective magnetic moment is proportional to the square root of the nanoparticle number in the MMC.

**Figure 27:**
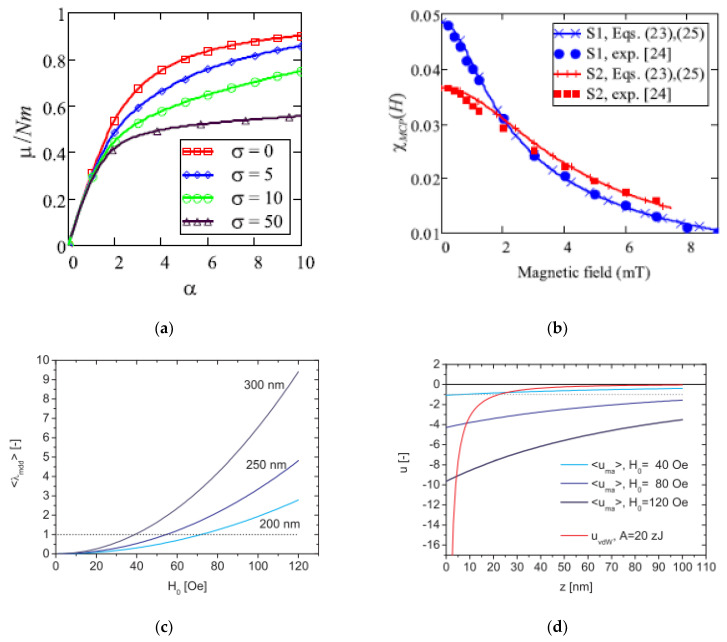
(**a**) Magnetic field dependence in Langevin units of the MMC magnetic moment for four values of the anisotropy constant. (**b**) Magnetic field dependence of MMC susceptibility: experiment and theoretical fit (Reprinted figure from [270]. Copyright 2020 by the American Physical Society), (**c**) Magnetic field amplitude dependence of the magnetic dipole–dipole interaction parameter, and (**d**) MMC surface separation dependence of van der Waals and magnetic dipole–dipole energies. (Reprinted from [271], Copyright 2020, with permission from Elsevier).

The static (DC) magnetic response of MMCs was theoretically calculated by Ivanov and Ludwig [270]. The MMC is composed of non-interacting, highly packed, and randomly oriented uniaxial magnetic nanoparticles. The model allows the computation of the magnetic field dependence of MMC’s magnetic moment (Figure 27a) and susceptibility. The fit of MMC susceptibility experimental data (Figure 27b) provides estimates of the constituent nanoparticles’ anisotropy constant and magnetic moment. Socoliuc and Turcu [271] calculated the low AC field dependence of the magnetic moment for 250 nm MMCs made from 8 nm magnetite nanoparticles, taking into consideration the influence of the demagnetizing field. The magnetic moment expression was used to compute the AC field dependence of the magnetic dipole–dipole energy in order to assess the colloidal stability of the MMG water dispersion (Figure 27c). It was found that in accordance with experimental data, the 250 nm MMGs in fields higher than 60 Oe lead to micron thick zippered chains that are tens to hundreds of microns long. The influence of the van der Waals interaction among MMCs was also investigated (Figure 27d).

**Figure 28:**
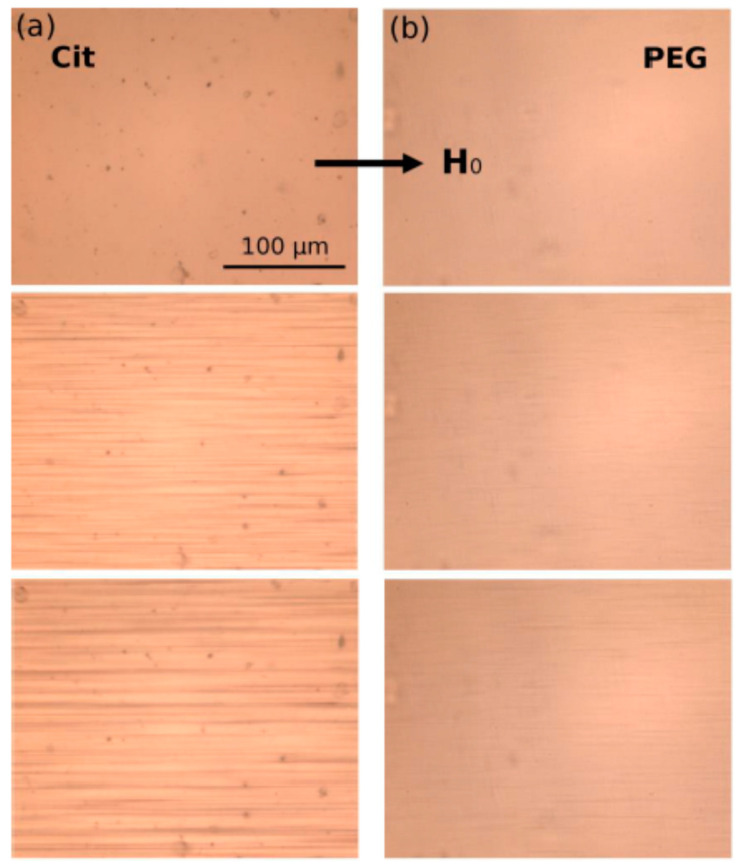
Optical microscopy images of aqueous suspensions of (**a**) citrated, and (**b**) PEGylated MNCs in an external uniform DC magnetic field of intensity 13.5 kA/m. Each row corresponds to the elapsed time from the moment of the magnetic field application *t* = 0 (upper row), 5 and 10 min. (Reprinted from [274] under Open Access license).

**Figure 29:**
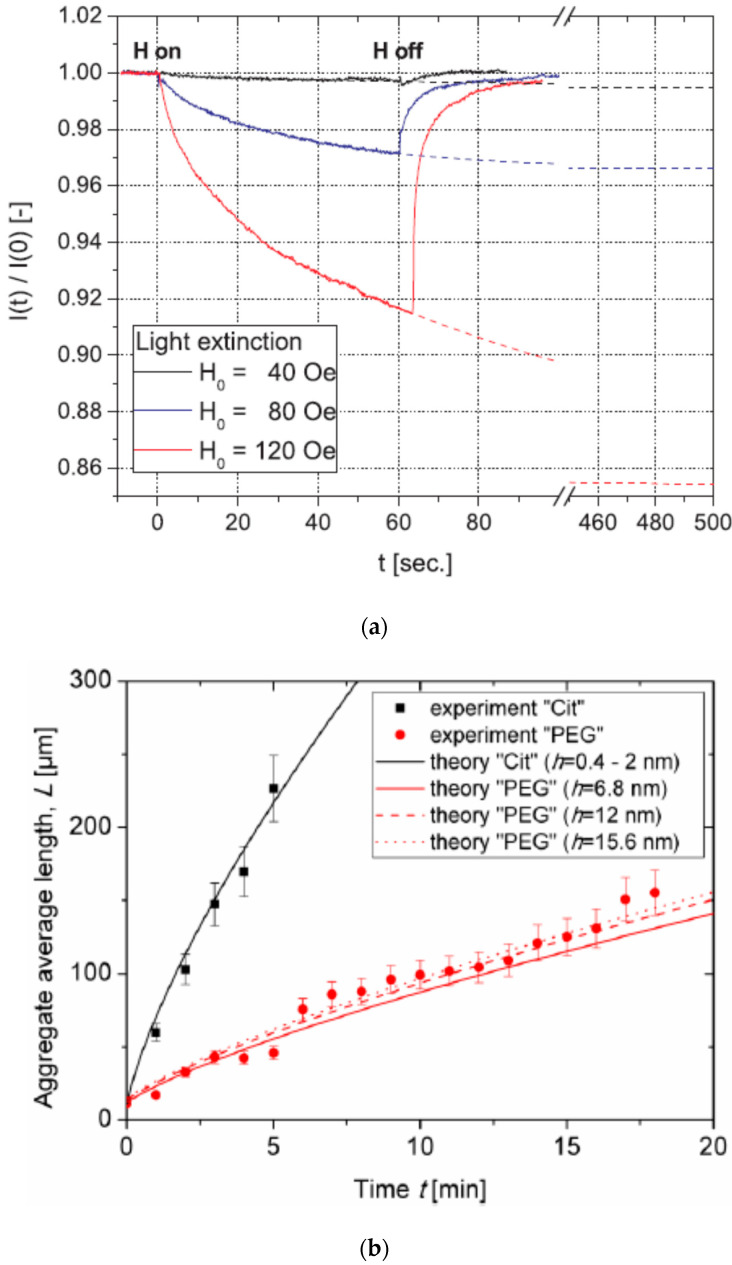
Kinetics of MMC magnetically induced clustering: (**a**) light extinction in 100 kHz AC magnetic field (reprinted from [271], Copyright 2020, with permission from Elsevier), and (**b**) optical microscopy in 170 Oe DC magnetic field (reprinted from [274] under Open Access license).

The magnetic moment is a crucial factor for understanding the spontaneous and magnetically induced clustering of MMCs colloids [272]. Spontaneous or magnetically induced, if the magnetic moment is large enough such that the attraction energy exceeds the thermal energy, MMCs will end up forming clusters whose shape and size depend on the field intensity and field exposure time. Once in contact due to magnetic attraction, the van der Waals attraction may prevent clusters disintegration after the field removal (Figure 27b). The MMC clusters morphology and formation kinetics were investigated both theoretically and experimentally (optical microscopy) [273,274], and static light scattering [271,273] was investigated as well. After external magnetic field application, about a micron thick and from tens up to a hundred microns-long spindle-like clusters begin to form, grow, and coalesce (Figure 28). The clustering process time scale may range up to tens of minutes, mainly depending on the applied magnetic field intensity (Figure 29). Socoliuc and Turcu have shown that MMC clustering also may occur in high-frequency AC magnetic fields [271]. The aggregation has a noticeable influence on the applicability of MMCs: it reduces MRI T2-weighted signal intensity [275] and significantly lowers the colloid-specific surface with a potential negative impact on drug targeting) [273,274] and hyperthermia [271] applications, not to mention the possibility of blood vessel clothing in vivo, which could be life-threatening. In the above context of particle clustering, it has to be mentioned that the adhesion of colloidal particles may not lead to a decrease in the specific surface area in aqueous media, since a hydrate layer, i.e., at least a water monolayer, is present on the particle surface. Particle collisions never cause dehydration, although the accessibility of surface sites in e.g., narrower pores may be kinetically hindered. Drying of aggregates, on the other hand, may cause an irreversible change to the MMCs, potentially reducing their applicability.

The collective interaction between constituent MNPs is a key feature in the MMCs in practical applications where an AC magnetic field excitation is involved, such as magnetic hyperthermia and MRI. Due to the high packing degree of the MNPs, the role of the dipolar interaction on the MMC magnetization dynamics must be related to the magnetic properties of the MNPs. Therefore, MMC design needs to take into account the particular magnetic properties of the constituent MNPs [260]. Numerical simulations carried out by Landi [276] showed that the dipolar interaction leads to SAR enhancement in the case of soft magnetic particles and SAR diminishing in the case of hard magnetic particles. On the experimental side, the large discrepancies reported in the literature regarding the MMC magnetic hyperthermia efficiency is discussed by Lartigue and coworkers [75]. Recent results concerning the magnetic hyperthermia performances of single- and multi-core magnetic particle systems designed for medical applications are presented and analyzed in [277,278], taking into account dipole–dipole and exchange interactions and also nonlinear field effects, evidencing the still existing differences in data acquisition and interpretation.

**Figure 30:**
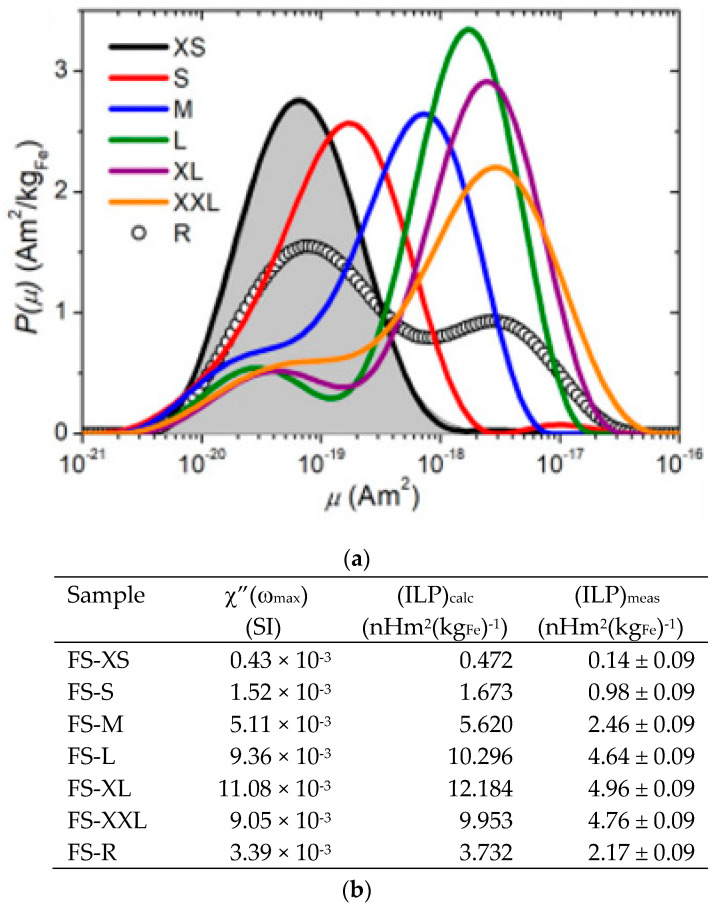
(**a**) FeraSpin MMC magnetic moment distributions determined from DC magnetization data: discrete moment-weighted apparent moment distributions P(µ) = M_sp_ (µ) Δµ of the colloids determined by numerical inversion of the M(H) curves. The gray area is the transformed and rescaled distribution calculated for a number-weighted lognormal distribution p(μ) with σ = 1.1 and a mean value of ‹µ› = 3.6 × 10^−20^ A m^2^ and (**b**) Intrinsic Loss Power of FerraSpin-R fractionated MMCs (republished with permission of IOP Publishing, from [279]; permission conveyed through Copyright Clearance Center, Inc.).

Bender and coworkers [279] investigated a series of colloids with fractionated FeraSpin-R MMCs from smallest to largest: -R, XS, S, M, L, XL, XXL. DC magnetization and optomagnetic measurements allowed for the determination of the MMC’s magnetic moment mono and bimodal distributions in the range 10^−20^–10^−16^ Am^2^ (Figure 30a). AC imaginary susceptibility and measured Intrinsic Loss Power (ILP) were found to increase with increasing MMC size and magnetic moment respectively, in the range 0.14–4.96 nHm^2^/kg_Fe_ (Figure 30b).

**Figure 31:**
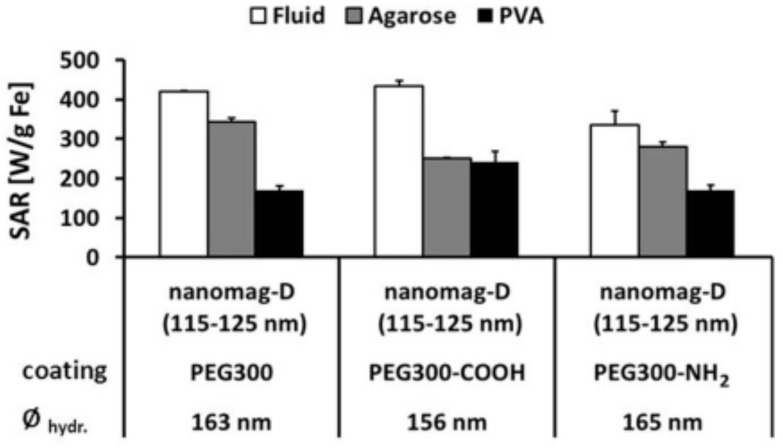
SAR diminishing after immobilization in agarose and PVA (reprinted from [280] under Open Access license).

A study regarding the influence of MMC mobility on hyperthermia efficiency was conducted by Ludwig and coworkers [280]. MMCs with diameters in the range 100–200 nm and different coatings (starch, PEG300, PEG300-COOH, and PEG300-NH2), dispersed in water and immobilized in 10% PVA gel and 1% agarose gel were characterized (DLS, DC magnetization, and magnetic relaxometry) and investigated. It was found that the Specific Absorption Ratio (SAR) diminishes after immobilization, which was more pronounced in the case of PVA than in the case of agarose, the former gel having smaller pores (Figure 31).

The role of the synergistic magnetism in MMCs is best outlined when their performance is compared with that of the constituent magnetic single core (MSC) nanoparticles. Four types of MMCs and constituent MSCs dispersed in water were investigated in [75] with respect to their MRI and magnetic hyperthermia efficiency. The MMCs and MSCs were characterized by means of TEM, DLS, DC magnetometry, ZFC/FC, and ferromagnetic resonance. ZFC/FC measurements revealed a drastic increase in the blocking temperature from MSC to MMC.

**Figure 32:**
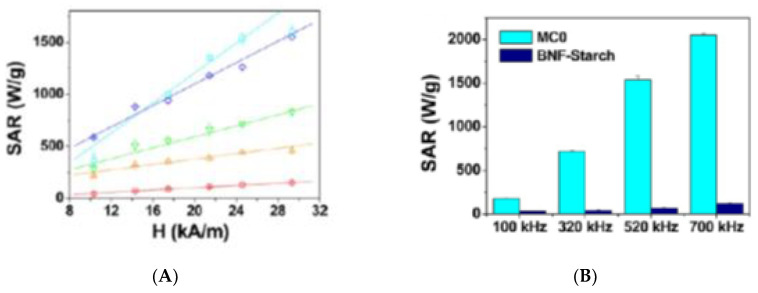

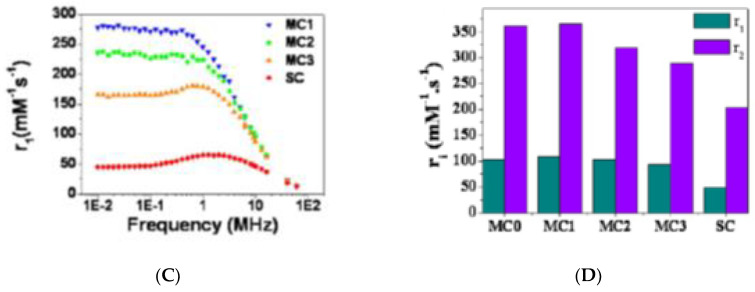
(**A**) Field amplitude dependence of the specific absorption ratio (SAR) for MMCs with decreasing diameter from MC0 to MC3 (MC0 (cyan), MC1 (blue), MC2 (green), MC3 (orange)) and magnetic single core nanoparticles (red), (**B**) SAR comparison between MC0 sample and commercial BNF starch for four frequency values, (**C**) Frequency dependence of r_1_ relaxivities, and (**D**) r_1_ and r_2_ relaxivities (reprinted with permission from [75]. Copyright 2012 American Chemical Society).

The MMCs, with decreasing diameter from MC0 to MC3, show decreasing SAR, but all of them were larger than that of the MCS (Figure 32A). The SAR of MC0 and MC1 is more than 500 times larger than that of MSC over the entire amplitude field range. Both the amplitude and the frequency dependence of the SAR show an unusual linear dependence (Figure 32A,B). The NMR performance of the multi-cores is also much better than that of the single cores both for spin-lattice (r_1_) and spin-spin (r_2_) relaxivities (Figure 32C,D).

## 6. Conclusions

Ferrofluids have proven to be an excellent primary nanomaterial in a large variety of magnetic nanoparticle assembly strategies that provide structural and morphological flexibility and functional adjustability in manufacturing multi-core magnetic composite particles. The architectural and functional diversity of the assembled multi-core magnetoresponsive particles with high magnetic response is devoted to meet the requirements of the most sophisticated applications in nanomedicine and biotechnology. The procedures applied, starting usually from easily evaporating and colloidally stable ferrofluids, facilitate a precise spatial organization of magnetic nanoparticles into spherical and a great diversity of non-spherical assemblies. The structure of individual particles as well as the organization into various assemblies can be followed with a combination of techniques (among others, electron and optical microscopy, small-angle neutron and X-ray scattering, magnetometry)—as described in this paper, thus allowing for detailed optimization of procedures and particle/assembly structure.

The great variety of magnetic multi-core particles manufactured using ferrofluids illustrate the progress in the design and production of these versatile magnetic vectors with adjustable physicochemical properties (core size, magnetic moment, surface charge, morphology, composition, and thickness of shell), taking into account the requirements of achievable magnetic field strength and gradient, as well as of colloidal stability in biorelevant media. Highly efficient ferrofluid-based manufacturing procedures provide a large variety of functionalized multi-core magnetic particles for nanomedicine (MRI contrast agents, magnetic drug targeting, magnetic field triggered drug release, hyperthermia, regenerative medicine, tissue engineering) and biotechnology (magnetic bioseparation, biosensors, protein immobilization, biocatalysis, heavy metal extraction/water purification, swimming nano- and microrobots).
